# Alpha-Lactalbumin Enriched Whey Protein Concentrate to Improve Gut, Immunity and Brain Development in Preterm Pigs

**DOI:** 10.3390/nu12010245

**Published:** 2020-01-17

**Authors:** Charlotte Holme Nielsen, Yan Hui, Duc Ninh Nguyen, Agnethe May Ahnfeldt, Douglas G. Burrin, Bolette Hartmann, Anne Birgitte Heckmann, Per Torp Sangild, Thomas Thymann, Stine Brandt Bering

**Affiliations:** 1 Department of Veterinary and Animal Sciences, Comparative Pediatrics and Nutrition, Faculty of Health and Medical Sciences, University of Copenhagen, 1870 Frederiksberg C, Denmark; charlotte.holme.nielsen@sund.ku.dk (C.H.N.); dnn@sund.ku.dk (D.N.N.); agnethe.ahnfeldt@sund.ku.dk (A.M.A.); pts@sund.ku.dk (P.T.S.); thomas.thymann@sund.ku.dk (T.T.); 2 Department of Food Science, Faculty of Science, University of Copenhagen, 1958 Frederiksberg C, Denmark; huiyan@food.ku.dk; 3 Department of Pediatrics, USDA-ARS Children’s Nutrition Research Center, Baylor College of Medicine, Houston, TX 77030, USA; doug.burrin@ARS.USDA.GOV; 4 Department of Biomedical Sciences and Novo Nordisk Foundation Center for Basic Metabolic Research, Faculty of Health and Medical Sciences, University of Copenhagen, 2200 Copenhagen, Denmark; bhartmann@sund.ku.dk; 5 Arla Foods Ingredients, 8260 Viby J, Denmark; anne.birgitte.lau.heckmann@arlafoods.com; 6 Department of Neonatology, Rigshospitalet, 2200 Copenhagen, Denmark

**Keywords:** alpha-lactalbumin, bioactive milk, preterm, growth, gut, immunity, cognition, microbiota, nutrition

## Abstract

Human milk is rich in nutritional factors, such as alpha-lactalbumin (α-Lac), and important for neonatal development, but nutrient supplementation may be required for optimal growth. Using a pig model, we hypothesized that α-Lac-enriched whey protein concentrate (WPC) supplementation improves neonatal development. Cesarean-delivered preterm pigs were fed either dilute bovine milk (REF) or REF milk supplemented with WPC with normal (STANDARD-ALPHA) or high (HIGH-ALPHA) α-Lac. Clinical, gut, immune and cognitive endpoints (open field, T-maze) were assessed and tissues collected at Day 19. The growth of STANDARD-ALPHA and HIGH-ALPHA were higher than REF (31 vs. 19 g/kg/d). Most organ weights, gut, immunity and brain variables were similar between WPC groups. HIGH-ALPHA had a higher bone mineral content, colon microbial diversity and an abundance of specific bacteria and microbial metabolites, and tended to show a faster food transit time (*p* = 0.07). Relative to REF, WPC pigs showed higher relative organ weights, blood amino acids, blood neutrophil function, and microbial metabolites, but lower brush-border enzyme activities and plasma cortisol. Cognition outcomes did not differ among the groups. In conclusion, WPC supplementation of milk improved some growth, gut and immunity parameters in preterm pigs. However, increasing the α-Lac content beyond human milk levels had limited effects on the immature gut and developing brain.

## 1. Introduction

Preterm birth (<37 weeks gestation) occurs in ~10% of all pregnancies worldwide [1], and preterm infants are predisposed to both short- and long-term complications [2], including feeding intolerance, infections, sepsis, necrotizing enterocolitis (NEC), and postnatal growth restriction [3,4,5]. The very preterm infants, born before 32 weeks gestation, particularly suffer from delayed neurodevelopment and cerebral defects [2,6,7,8]. Human milk improves clinical outcomes by providing nutrients and bioactive and anti-inflammatory factors [5,9]. In cases where the mother’s own milk or donor human milk is limited or unavailable, there may be a need to provide infant formulas or fortifiers with high quality protein to secure proper growth.

It is of interest to develop infant formulas with a protein and amino acid profile more similar to human milk to improve growth and gastrointestinal tolerance [10]. Alpha-lactalbumin (α-Lac) is the predominant whey protein in human milk and shares high amino acid sequence homology with bovine α-Lac (~72%) [11,12]. This accounts for approximately 25% of the total protein in human milk, whereas in bovine milk it accounts for only 3.5%, due to the higher casein to whey ratio. The concentration is 2.9 g/L in mature human milk, and about 3.5 g/L in transitional milk [13]. Further, α-Lac provides essential amino acids for protein synthesis in the developing neonate, particularly tryptophan, which is one of the limiting amino acids for growth in infant formulas [10], and also a precursor of serotonin and melatonin [14]. Supplementation with α-Lac in adults has been shown to enhance brain tryptophan and serotonin levels, which are associated with improved cognition in stress-vulnerable individuals [15], and better memory [16] and sleep [17]. Studies in healthy-term infants have shown that a low-protein formula with a high proportion of α-Lac provided similar plasma tryptophan concentrations, gastrointestinal tolerance and growth relative to human milk-fed infants [10,18,19]. Similar observations are seen in infant rhesus monkeys [20]. The bioactive properties of α-Lac, and peptides released from α-Lac during digestion, relate to antimicrobial activity [21,22], prebiotic properties [22,23,24] and epithelial restoration via selective apoptotic activity [25]. Particularly the growth-promoting properties of α-Lac on several *Bifidobacterium* strains [24] may push the microbiome of formula-fed infants towards a pattern more similar to human milk-fed infants. The antibacterial peptides released from α-Lac during digestion may also exert immunostimulatory effects by inducing phagocytic activity, as shown in human and murine macrophages [26]. The α-Lac may therefore be an important protein ingredient in infant formulas for improved growth, gut microbiota, immunomodulation and brain development. This is particularly important in newborn preterm infants with compromised organ functions.

Preterm pigs born at 90% gestation have close similarities to preterm infants with regards to organ development, clinical complications, postnatal adaptation and delayed neurodevelopment [27,28,29]. Like humans, pigs show pre- and postnatal brain growth spurts [30], suggesting that the pig brain may also be vulnerable to complications related to preterm birth. Further, their high sensitivity to dietary feeding makes the preterm pig an excellent model to study dietary interventions and effects on postnatal maturation and development [28]. Given the potential effects on several organ systems, we hypothesized that supplementation with a bovine whey protein concentrate (WPC), with or without α-Lac enrichment (e.g., 1–4-fold of levels in transitional human milk), would improve growth, gut function, microbiota, immunity, and brain structure and function. We tested this in preterm pigs as a model of newborn infants.

## 2. Materials and Methods

### 2.1. Animals and Experimental Design

All experimental animal procedures were approved by Danish Animal Experiments Inspectorate (protocol no. 2014-15-0201-00418) in accordance with the guidelines from Directive 2010/63/EU of the European Parliament and the Animal Research: Reporting of In Vivo Experiments (ARRIVE) Guidelines [31]. Forty neonatal pigs (Danish Landrace × Large White × Duroc) were delivered by cesarean section at 90% gestation (106 days) from two sows, as earlier outlined [28,29]. All pigs were resuscitated and placed in heated (37–38 °C) and oxygenated incubators. The pigs were block randomized according to birth weight and gender to two treatment groups receiving diets consisting of basal bovine milk supplemented with a bovine WPC enriched with α-Lac (HIGH-ALPHA, *n* = 19), or basal bovine milk supplemented with a bovine WPC with a standard α-Lac content (STANDARD-ALPHA, *n* = 20). As a reference, we included pigs from two separate litters that received the same basal bovine milk diet without WPC supplementation (REF, *n* = 18). Data from the REF pigs were derived from a previous study (Ahnfeldt, A.M.; Bæk, O. et al. Nutrient restriction has limited short-term effects on gut, immunity and brain development in preterm pigs. *J. Nutr.* (under review)).

All pigs were reared in individual incubators, and were fitted with a vascular catheter (4F, Portex, Kent, UK) inserted into the umbilical artery for parenteral nutrition (PN) and blood sampling. Further, an oro-gastric feeding tube (6F, Portex) was inserted for enteral feeding, as previously described [28]. To provide passive immunization, sterile maternal plasma (25 mL/kg) was infused through the vascular catheter within the first 24 h after birth [28].

### 2.2. Diets and Nutrition

Each pig was weighed daily and, during days 1–7, the pigs received continuous PN at doses decreasing from 120 mL/(kg·day) on Day 1 to 48 mL/(kg·day) on Day 7. The PN solution (Kabiven, Soluvit, Vitalipid, Vamin Fresenius Kabi, Bad Homburg, Germany) was adjusted to meet the requirements of preterm pigs [28,29].

The pigs were fed increasing amounts of enteral nutrition (EN) (32–180 mL/(kg·day)). Both the HIGH-ALPHA, the STANDARD-ALPHA and the REF diet consisted of a base of raw unpasteurized bovine milk diluted 2:1 with water and with added minerals and vitamins (Paediatric Seravit, 15 g/L, Nutricia, Allerød, Denmark and Revolyte Nutrition, 6 g/L, Gunnar Kjems ApS, Copenhagen, Denmark). For the HIGH-ALPHA diet, the diluted milk was fortified with an α-Lac-enriched bovine WPC (Arla Foods Ingredients Group P/S, Viby J, Denmark), resulting in a final α-lac concentration of 18 g/L. This corresponded to HIGH-ALPHA pigs receiving 2.7–3.2 g α-Lac per kg body weight per day from birth until the end of the study period. For the STANDARD-ALPHA diet, diluted milk was fortified with a WPC with a standard α-Lac content (Arla Foods Ingredients Group P/S), resulting in a final α-Lac concentration of 6.3 g/L, corresponding to an intake of 0.96–1.1 g α-Lac per kg body weight per day in the STANDARD-ALPHA group. Compared with normal porcine milk [32], the dilute REF diet had a lower content of all macronutrients, i.e., −44% energy, −51% protein, −44% carbohydrate, and −42% fat. The nutrient contents and osmolality of all three diets are listed in Table 1. The osmolality was measured using a cryoscopic osmometer (Osmomat, Gonotec, Berlin, Germany) [33]. The diets were prepared on a daily basis and kept at 4 °C, and the personnel were blinded to the treatments throughout the experiment. The pigs were fed every second hour during the day and every third hour during the night until Day 9, followed by feedings every third hour. During the first five days, pigs were fed via the oro-gastric tubes, followed by transition to voluntary feeding from troughs. From Day 11, all pigs had ad libitum access to water, and on Day 12, the oro-gastric tubes were removed.

### 2.3. Clinical Evaluation and Animal Procedures

The clinical condition of the pigs was evaluated from assessment criteria and clinical scores were recorded twice daily with scores ranging from 1 (healthy), 2 (mild symptoms), 3 (moderate symptoms) to 4 (severe symptoms). Assessment criteria included pain, lethargy and reduced interest in surroundings, weakness, paleness and cyanosis, skin changes, cold extremities and reduced appetite. If signs of pain were seen, either meloxicam (Metacam, 5 mg/mL, Boehringer-Ingelheim, Copenhagen, Denmark) or butorphanol (Torbugesic, 10 mg/mL; Scanvet, Fredensborg, Denmark) was administered intramuscularly. Fecal scores were recorded twice daily as: 1 (firm feces), 2 (pasty feces), 3 (droplets of watery feces), 4 (moderate amounts of diarrhea) to 5 (extensive diarrhea). In cases of diarrhea, oral electrolyte supplementation (Revolyte Nutrition, 2–5 mL, Gunnar Kjems) was provided to avoid dehydration. Prophylactic oral antibiotics were administered twice daily from day 8–10 (gentamicin: Gentocin Vet., 4.35 mg/mL, Scanvet; amoxicillin, 50 mg/mL, with clavulanic acid, 12.5 mg/mL: 2care4, Esbjerg, Denmark; metronidazole: Flagyl, 40 mg/mL, Sanofi-Aventis, Hørsholm, Denmark).

### 2.4. Body Composition and Tissue Collection

On Day 19, all pigs were anaesthetized with an intramuscular injection of zolazepam/tiletamin (Zoletil 50, Virbac, Kolding, Denmark), xylazine (Xysol, Scanvet), ketamine (Ketaminol, MSD Animal Health, Copenhagen, Denmark) and butorphanol (Torbugesic, Scanvet). The body composition of each pig was measured in ventral recumbency by Dual Energy X-ray Absorptiometry (DEXA, Lunar Prodigy scanner, GE Healthcare, Little Chalfont, UK). While still anaesthetized, a blood sample was drawn, and pigs were subsequently euthanized with an intracardial injection of pentobarbital. The brain and abdominal organs were dissected and weighed, and samples were collected and frozen in liquid nitrogen and stored at −80 °C or fixed in 4% formaldehyde.

### 2.5. Blood Biochemistry and Blood Analyses

Blood was collected one hour after feeding on Days 8 and 19 and centrifuged (2500× *g*, 10 min, 4 °C). Isolated serum was analyzed for biochemical profile (Siemens ADVIA 1800 Chemistry System, Siemens Healthcare A/S, Ballerup, Denmark), while isolated plasma was used for analysis of postprandial plasma free amino acid profile. Plasma for the amino acid profile was analyzed using reverse-phase High-Performance Liquid Chromatography [34]. Plasma cortisol, insulin-like growth factor (IGF-1) and C-reactive protein (CRP) were measured using a commercial porcine enzyme-linked immunosorbent assay (ELISA) kit according to the manufacturer’s protocols (Porcine Duoset, R&D Systems, Abingdon, UK). The plasma concentration of serotonin on Day 19 was quantified using a commercial ELISA kit (Serotonin ELISA, IBL International GmbH, Hamburg, Germany).

### 2.6. Hematology and Systemic Immunity

Blood for hematology, fluorescence-activated cell sorting (FACS), T-cell characterization and evaluation of neutrophil phagocytic function was collected at birth, on Day 8 and on Day 19 in EDTA tubes. For analysis of hematology, an automatic cell counter (Advia 2120i Hematology System, Siemens, Germany) was used. Following lysis and staining of the lymphocytes, performed as previously described with minor modifications [35], the T-cell subsets were determined by flow cytometry (BD Accuri C6 flow cytometer, BD Biosciences). T-cell subsets were defined as T-cells (CD3^+^ lymphocytes), helper T-cells (CD3^+^CD4^+^CD8^−^ lymphocytes) and cytotoxic T-cells (CD3^+^CD4^−^CD8^+^ lymphocytes).

Neutrophil phagocytic function was measured in whole blood using pHRodo-Red conjugated *Escherichia coli* bioparticle phagocytosis kit for flow cytometry (ThermoFisher, Roskilde, Denmark) [36]. The phagocytic rate of neutrophils was defined as the percentage of neutrophils with engulfed bacteria, and the phagocytic capacity as the median fluorescent intensity (MFI) of neutrophil population with internalized bacteria.

### 2.7. Gut Morphology

To measure villus heights and crypt depths in the intestinal mucosa of proximal, mid and distal small intestine, tissue sections were paraffin-embedded, sliced and scanned using arrayWoRx microarray scanner (Applied Precision, Issaquah, WA, USA) and the morphometric software softWoRx Explorer 1.1 (Applied Precision) [37]. Goblet cell density was counted (STEPanizer stereology tool, version 1.0) in samples from colon and distal small intestines from images obtained at 200× magnification by light microscope (Olympus BX45TF, Tokyo, Japan) and a camera with belonging software, cell^A (version 3.4, Olympus).

### 2.8. Gut Function

Intestinal lactose digestive capacity was assessed on Day 5 by oral administration of a 10% lactose solution at a dose of 15 mL/kg [38]. Blood was sampled prior to administration and the increase in blood galactose concentration was measured after 40 min. Gastric emptying was investigated on Day 19 by adding 10 mg/mL acetaminophen to the enteral milk diet one hour before euthanasia. The concentration of acetaminophen in cardiac blood just prior to euthanasia was measured. On Days 3, 10 and 19, all pigs received chromium oxide (CR_2_O_3_) added to the enteral milk diets at 3 mg/mL, to assess total gastrointestinal transit time by recording the number of hours before the feces turned green. On Day 19, the harvested intestinal sections were further evaluated for the accumulation of chromium oxide one hour after administration.

Concentrations of glucagon-like peptide-1 (GLP-1) and insulin were measured in plasma on Day 19. All samples were extracted in 70% ethanol before GLP-1 measurement. Total GLP-1 was measured [39] using a radioimmunoassay specific for the C-terminal of the GLP-1 molecule (antibody code no 89390) and reacting equally with intact GLP-1 and the primary (N-terminally truncated) metabolite. Sensitivity was below 1 pmol/L, and the intra assay coefficient of variation was below 10%. Insulin was measured in duplicates using a porcine insulin ELISA kit (cat no 10-1200-01; Mercodia, Sweden) according to the manufacturer’s instructions. Measurement range was 2.3–173 mU/L.

Activities of the brush-border disaccharidases (lactase, sucrase and maltase) and peptidases (aminopeptidase N (ApN), aminopeptidase A (ApA), and dipeptidyl peptidase 4 (DPP4)) were measured in tissue homogenates from the proximal, mid and distal small intestine by spectrophotometry [40].

### 2.9. Microbiome and Microbial Metabolites

To semi-quantify bacterial abundance in the intestinal lumen and tissue, fluorescent in-situ hybridization (FISH) was performed on sections from distal small intestines, using an eubacterial fluorescent-labelled DNA-probe (Eub H456, 39-3210-1/1, sequence: 5′-[AF 555]GCTGCCTCCCGTAGGAGT-3′, Eurofins, Glostrup, Denmark) matching bacterial DNA. The specific experimental procedure was outlined previously [41]. Bacterial micro-colonies were visualized microscopically (Leica Leitz DMRB, Leica Microsystems, Brønshøj, Denmark) and images were obtained using a microscope camera (Monochrome digital camera for fluorescence applications, Leica DFC350 FX, Leica Microsystems). Based on the red fluorescence signal expressing bacterial abundance, tissue slides were assigned a standardized FISH-score from 1 (no/very few on epithelium), 2 or 3 (few on epithelium, locally or globally), 4 or 5 (medium abundance on epithelium, locally or globally), 6 or 7 (larger abundance on epithelium, locally or globally) to 8 or 9 (invasion in intestinal tissue, locally or globally).

Gut microbiota were evaluated using total DNA extracted from the colon content collected at euthanasia on Day 19 and the composition was determined by 16S rRNA bacterial gene V3 region amplicon through NextSeq PE150 amplicon sequencing (Illumina, San Diego, CA, USA), as described previously [42]. The raw sequencing reads were merged and trimmed, chimeras removed and zero-radius Operational Taxonomic Units (zOTUs) constructed using UNOISE algorithm, implemented in Vsearch [43]. Greengenes (version 13.8) database was used as a reference for annotation. Total observed zOTU numbers and Shannon index in each respective sample were calculated for alpha diversity comparison after minimum sample depth (7120) rare fraction. The raw OTU table was normalized with cumulative sum scaling (CSS) [44] to calculate the binary Jaccard and Bray Curtis distance for principle coordinate analysis (PCoA) visualization.

Concentrations of microbial metabolites and short chain fatty acids (SCFAs) in colon contents (acetic-, butanoic-, propanoic-, valeric-, 3-methyl butanoic-, hexanoic- and isohexanoic acid and propanediol), were measured by gas chromatography-mass spectroscopy (GC-MS) and data presented as µmol/g of wet matter [45]. Lactate concentrations were measured using an Agilent LC/MS 1100 system [46].

### 2.10. Physical Activity, Behavior and Cognition

As basic motor skills, the acquisition of first eyelid opening, first stand, first walk and number of hours spent by each pig learning to drink a full bolus of milk were recorded for each pig [47].

Physical activity was measured continuously in incubators (Day 1–5) and larger cages (Day 5–10) using a motion-sensitive, infrared surveillance camera placed over each incubator [29,47]. The digital output was analyzed as activity bouts and the proportion of active time in one hour periods using PIGLWin software program (Ellegaard Systems, Faaborg, Denmark) [29]. Data analysis started 12 h after the cesarean section and, as a lower threshold of a positive activity, a filter of 5 sec was applied post hoc when retrieving data [47].

Free movement behavior was investigated on Day 9 in an open field arena (1.2 × 1.2 m). In a randomized order, each pig was camera-recorded from a bird’s eye view while walking freely for 3 min [29,47]. Travelled distance and velocity were analyzed using EthoVision XT10 software (Noldus Information Technology, Wageningen, The Netherlands).

Following two days of habituation, memory and spatial orientation were tested in a plus-shaped T-maze during Days 13–18, as previously described [48]. In brief, the pigs were placed in a starting position and navigated by visible extra-maze cues to locate a milk reward. An acquisition included ten trials and each pig conducted one to two acquisitions per day. In the forward phase from Acquisition 3–6, the milk reward was placed in the same arm with alternating start positions, whereas it was switched to the opposite arm in the reversal phase in Acquisition 7–9. The learning criterion was defined as a minimum of 80% correct choices out of ten trials. Each trial was camera-recorded from a bird’s eye view and all camera-recordings were analyzed using Ethovision XT10 software (Noldus Information Technology).

### 2.11. Statistics

Data analyses were performed using the software R (version 3.4.3, The R Foundation for Statistical Computing, Vienna, Austria). All data analyses for HIGH-ALPHA and STANDARD-ALPHA were adjusted for diet, birth weight and gender as fixed variables and litter as a random variable. Continuous data were analyzed by linear mixed models (lm function, lme4 package). Data from repeated measurements (daily body weight, T-maze and physical activity) were analyzed by linear mixed models with repeated measures (lmer function, nlme package). Time for acquisition of basic motor skills was evaluated using a Mantel-Cox comparison between Kaplan–Meier survival curves by means of cox mixed-effects model and survfit function (coxme package, bdsmatrix package and survival package). Ordinal data (distal accumulation of chromium oxide in the gut and FISH) were analyzed by proportional odds logistic regression (polr function, MASS package and ordinal package). All data analyses for WPC versus REF were performed in a similar way, adjusting for diet, birth weight and gender. Litter was excluded as a random variable because the REF was derived from two separate litters of pigs.

Statistics on microbial data included Qiime (version 1.9.1) [49] for the calculation of alpha diversity combined with the R packages Vegan, rstatix and ggplot2 to process post-analysis and visualization. Wilcoxon signed rank test (rstatix package) was used to analyze differences in alpha diversity. To evaluate beta diversity differences, adonis (Vegan package) was performed. Litter effect was regarded as a condition effect in the constrained analysis based on Bray Curtis distance metrics. Specific taxa comparisons among groups were analyzed by ANCOM [50]. The default FDR-adjusted W value (*p* threshold = 0.05) in ANCOM was used to find statistically significant bacteria between groups. Pearson’s correlation analysis (Rhea package) was conducted between centered log-ratio transformed relative abundances of genera and the phenotype data. Pearson’s correlation matrix was plotted in a heat map. The Benjamini–Hochberg method was used to calculate adjusted *p*-values for multiple testing.

For all data, residuals and fitted values were assessed for normality and variance homogeneity to confirm model validity, and logarithmic data transformation was performed if required to meet normality and homoscedasticity assumptions. To correct for multiple comparisons between groups, Tukey or Dunnett corrections were used. Data are presented as means with standard deviations (SD), unless otherwise stated. The *p*-values < 0.05 were regarded as statistically significant and *p*-values ≤ 0.10 as a tendency to an effect.

## 3. Results

### 3.1. Clinical Characteristics and Growth

All HIGH-ALPHA and STANDARD-ALPHA pigs were healthy throughout the 19 days of the experiment, with stable physiological parameters and clinical scores of 1–2. From Day 14–19, few pigs had fecal scores of 3–4, but no pigs showed clinical signs of infection or inflammation. Of a total of 40 pigs, one pig was euthanized shortly after delivery due to extremely low birth weight and four pigs were euthanized during the course of the experiment because of poor clinical condition unrelated to diet (thrombus from intravascular catheter). Birth weights were similar between HIGH-ALPHA (832 ± 161 g) and STANDARD-ALPHA (811 ± 166 g), and body weights remained similar throughout the 19 days. The daily weight gain tended to be lower for HIGH-ALPHA versus STANDARD-ALPHA pigs (29.31 ± 5.27 versus 33.46 ± 5.29 g/(kg·day), *p* < 0.08, Figure S1A,B, Supplementary Materials).

**Figure 1:**
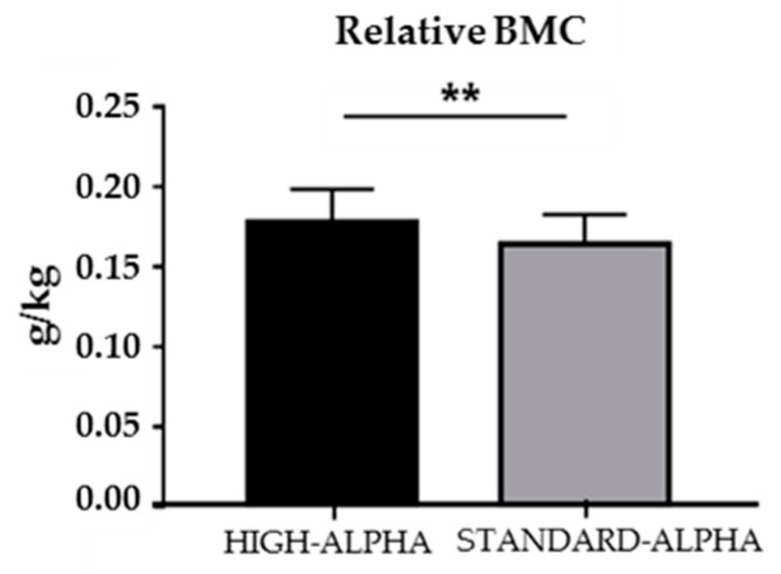
Dual-energy X-ray absorptiometry (DEXA) scans of 19 day old preterm pigs fed a bovine milk diet supplemented with bovine whey protein concentrate either enriched with α-Lac (HIGH-ALPHA, *n* = 16) or with standard content of α-Lac (STANDARD-ALPHA, *n* = 17). Relative bone mineral content (BMC). Data are expressed as mean ± SD. Significant differences between groups are shown (** *p* < 0.01).

Total bone mass density and relative bone mineral content on Day 19 were higher in HIGH-ALPHA compared to STANDARD-ALPHA pigs (0.20 ± 0.01 versus 0.19 ± 0.01 g/cm^2^, *p* < 0.05 and 17.82 ± 2.05 versus 16.41 ± 1.86 g/kg, *p* < 0.01, respectively, Figure S2A, Supplementary Materials and Figure 1). Lean mass and fat percentage were equal between groups (Figure S2B,C, Supplementary Materials).

Several REF pigs presented with bloody diarrhea and ileus was identified by abdominal ultrasound examination. Postmortem intestinal examination showed macroscopic signs of NEC. Additionally, several pigs experienced seizures (Ahnfeldt, A.M.; Bæk, O. et al. Nutrient restriction has limited short-term effects on gut, immunity and brain development in preterm pigs. *J. Nutr.* (under review)). None of these clinical complications were observed in the group of WPC-supplemented pigs (WPC). Compared with the pooled birth weights of the WPC pigs, the REF pigs were larger at birth (1032 ± 228 versus 822 ± 161 g, *p* < 0.001), but their daily weight gain was lower (19.2 ± 3.20 versus 31.33 ± 5.61 g/(kg·day), *p* < 0.001). Both WPC and REF pigs had comparable body weights at Day 19 (1464 ± 341 versus 1524 ± 314 g).

### 3.2. Organ Weights

The organ weights are given in Table 2. In general, both absolute and relative organ weights were similar between STANDARD-ALPHA and HIGH-ALPHA, albeit with a higher relative weight of the small intestine in STANDARD-ALPHA (Table 2, *p* < 0.05). Further, the absolute weights of kidneys tended to be higher in STANDARD-ALPHA (Table 2, *p* < 0.08), whereas the relative weights of adrenals tended to be higher in HIGH-ALPHA (Table 2, *p* < 0.08).

In WPC compared with REF pigs (Table S2, Supplementary Materials), the relative weights of the total small intestine, colon, liver and spleen were higher (all *p* < 0.01) and only the relative weights of the heart were lower in WPC (*p* < 0.05). Finally, the relative weights of the hippocampus and striatum were higher in WPC (*p* < 0.01 and *p* < 0.001), whereas the cerebral water content was lower compared with REF (*p* < 0.05).

### 3.3. Biochemistry, Amino Acid Profile and Blood Analyses

In general, the serum biochemical profile, as listed in Table S3, Supplementary Materials, was similar between HIGH-ALPHA and STANDARD-ALPHA. Exceptions on Day 8 included higher serum albumin (*p* < 0.05), creatine kinase (*p* < 0.05) and blood urea nitrogen (BUN, *p* < 0.001) in HIGH-ALPHA relative to STANDARD-ALPHA (Table S3, Supplementary Materials), while, on Day 19, serum creatinine and potassium levels were higher in HIGH-ALPHA relative to STANDARD-ALPHA (Table S3, Supplementary Materials, *p* < 0.05).

**Figure 2:**
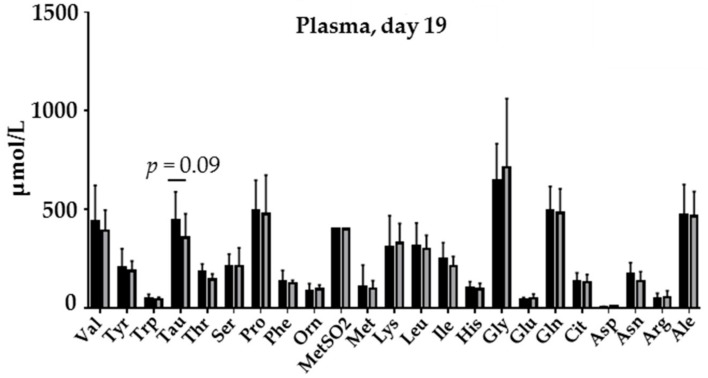
Plasma concentrations of amino acids in preterm pigs fed a bovine milk diet supplemented with bovine whey protein concentrate either enriched with α-Lac (HIGH-ALPHA, *n* = 15) or with standard content of α-Lac (STANDARD-ALPHA, *n* = 15) measured in blood samples at Day 19. Data are expressed as mean ± SD. *p* = 0.09 indicates a tendency of a difference between groups.

Whereas the amino acid profiles of the HIGH-ALPHA and STANDARD-ALPHA milk diets were similar (Figure S3A, Supplementary Materials), the plasma amino acid profile measured on Day 8 showed higher levels of valine, tryptophan, phenylalanine, isoleucine (*p* < 0.05, Figure S3B, Supplementary Materials) in the HIGH-ALPHA versus STANDARD-ALPHA. Only glutamic acid was higher in STANDARD-ALPHA (*p* < 0.05). On Day 19, the plasma amino acids were similar between HIGH-ALPHA and STANDARD-ALPHA (Figure 2).

General protein supplementation, as indicated by the comparison of the pooled WPC group relative to REF (Table S2, Supplementary Materials), resulted (on Day 19) in higher serum levels of albumin, total protein, phosphate, BUN, calcium, magnesium and sodium (*p* < 0.01), whereas BASP, cholesterol and iron were lower than in REF pigs (*p* < 0.05). Likewise, the WPC group had higher plasma levels in a range of both essential and non-essential amino acids on both Day 8 and Day 19 (Table S2, Supplementary Materials). Of the hormones measured, only cortisol levels were lower in WPC (*p* < 0.01).

### 3.4. Hematology and Systemic Immunity

The number of total leukocytes and lymphocyte subsets (lymphocytes, T-cells, helper T-cells (CD4^+^ cells) and cytotoxic T-cells (CD8^+^ cells)) was similar between HIGH-ALPHA and STANDARD-ALPHA at all time points (Table S4, Supplementary Materials). Likewise, immune cell counts (neutrophils, monocytes, eosinophils and basophils) were similar. Only mean platelet volume (MPV) was consistently higher in HIGH-ALPHA pigs (*p* < 0.05). There were no differences between HIGH-ALPHA and STANDARD-ALPHA in the remaining hematological and immunological parameters (Table S4, Supplementary Materials). Likewise, the neutrophil phagocytic rate and capacity was equal between HIGH-ALPHA and STANDARD-ALPHA throughout the study (Figure S4A,B, Supplementary Materials).

### 3.5. Gut Structure and Function

**Figure 3:**
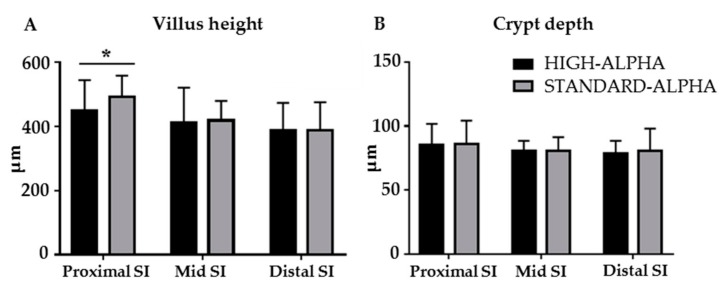
Structural gut endpoints measured in 19 day old preterm pigs fed a bovine milk diet supplemented with bovine whey protein concentrate, either enriched with α-Lac (HIGH-ALPHA, *n* = 15–18) or with standard content of α-Lac (STANDARD-ALPHA, *n* = 13–17). (**A**) Villus height and (**B**) Crypt depth. Data are expressed as mean ± SD. Significant differences between groups are shown (* *p* < 0.05).

In the STANDARD-ALPHA pigs, the villi in the proximal small intestine were higher on Day 19 than in HIGH-ALPHA pigs (493 ± 65.47 µm versus 448 ± 96.27 µm, *p* < 0.05, Figure 3A), whereas villus heights and crypt depths in all other regions were similar between the two groups (Figure 3A,B). Likewise, the density of the mucin-containing goblet cells in the distal small intestine and colon were similar (Figure S5A, Supplementary Materials). Moreover, activities of brush-border enzymes and lactose digestive capacity (Figure S5B,C, Supplementary Materials) were similar for STANDARD- and HIGH-ALPHA. The gastric emptying, measured as acetaminophen absorption on Day 19, in HIGH-ALPHA and STANDARD-ALPHA was similar between groups (Figure S5D, Supplementary Materials).

In WPC versus REF pigs (Table S2, Supplementary Materials), villi in the proximal small intestine and crypt depths in all parts of the small intestine were longer (*p* < 0.05), whereas the goblet cell density was lower on Day 19 (*p* < 0.01). The activities of sucrase, aminopeptidase A and dipeptidyl peptidase 4 were lower in WPC pigs (*p* < 0.01), while the intestinal hexose absorption and lactose digestive capacity were higher on Day 5 (*p* < 0.05).

Gastric emptying by chromium oxide test on Days 3 and 10 was similar between HIGH-ALPHA and STANDARD-ALPHA pigs (Figure S6A,B, Supplementary Materials). On Day 19, chromium oxide tended to reach the distal intestine in a higher number of HIGH-ALPHA pigs (*p* < 0.07, Figure S6C, Supplementary Materials). Plasma concentrations of GLP-1 on Day 19 were similar between HIGH-ALPHA and STANDARD-ALPHA, but insulin concentrations tended to be higher in STANDARD-ALPHA (*p* < 0.07, Table S3, Supplementary Materials). In WPC and REF, plasma concentrations of insulin and GLP-1 were similar (Table S2, Supplementary Materials).

### 3.6. Microbiome and Microbial Metabolites

**Figure 4:**
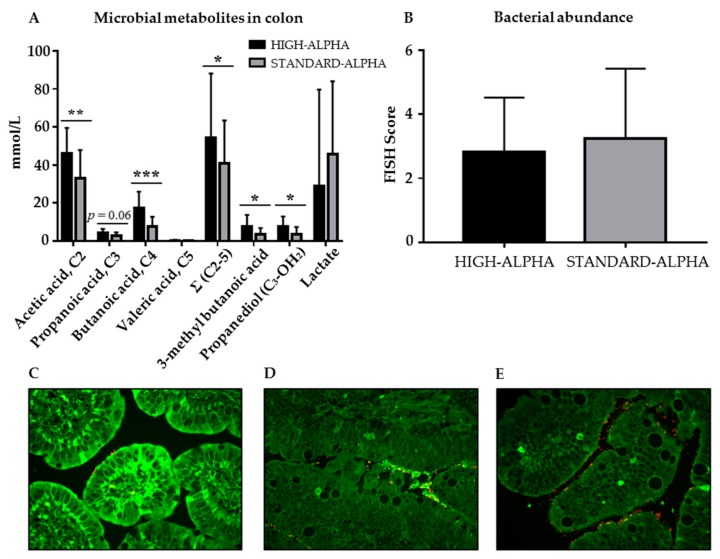
(**A**) Concentrations of microbial metabolites, including short chain fatty acids, in the colon of 19 day old preterm pigs fed a bovine milk diet supplemented with bovine whey protein concentrate, either enriched with α-Lac (HIGH-ALPHA, *n* = 16) or with standard content of α-Lac (STANDARD-ALPHA, *n* = 16). (**B**) Bacterial abundance in the tissue and lumen of distal small intestines based on FISH score. Microscopic visualization of distal small intestines from preterm pig diet groups with bacterial micro-colonies (HIGH-APLHA or STANDARD-ALPHA). Images representative of the standardized FISH-score interval of the means of both diet groups based on a fluorescent signal expressing the density of bacteria. (**C**) Score 2 (few bacteria on epithelium, locally); (**D**) score 3 (few bacteria on epithelium, globally) and (**E**) score 4 (medium abundance of bacteria on epithelium, locally). Data are expressed as mean ± SD. *p* = 0.06 indicates a tendency of difference between groups. Significant differences between groups are shown (* *p* < 0.05; ** *p* < 0.01; *** *p* < 0.001).

For microbial metabolites and SCFAs in colon contents on Day 19, HIGH-ALPHA had higher concentrations of total SCFAs and the individual acetic acid, butanoic acid, 3-methyl butanoic acid and propanediol relative to STANDARD-ALPHA (*p* < 0.05, Figure 4A). In WPC pigs, the concentrations of acetic acid and lactate, as well as the sum of SCFAs, were higher than REF (*p* < 0.05, Table S2, Supplementary Materials). FISH analysis showed similar bacterial abundance scores in HIGH-ALPHA and STANDARD-ALPHA (2.8 ± 1.7 versus 3.2 ± 2.2, respectively, Figure 4B). Images representative of the mean FISH-score interval of HIGH-ALPHA and STANDARD-ALPHA are presented in Figure 4C–E.

**Figure 5:**
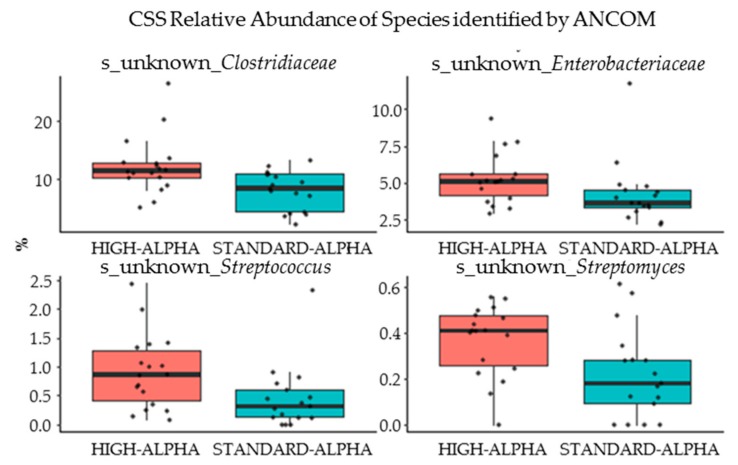
Gut microbiota determined by the 16s rRNA gene amplicon sequencing at Day 19 in preterm pigs fed a bovine milk diet supplemented with bovine whey protein concentrate either enriched with α-Lac (HIGH-ALPHA, *n* = 18) or with standard content of α-Lac (STANDARD-ALPHA, *n* = 17). Cumulative sum scaling (CSS) relative abundance of significantly differentially expressed bacterial genera in the colon, as determined by ANCOM.

A total of 20,482 ± 7896 high-quality reads per gut content sample from HIGH-ALPHA and STANDARD-ALPHA pigs were obtained and aligned to 780 zOTUs, of which 45 bacterial groups were classified at the genus level. The observed OTUs per sample, as a measure of alpha diversity expressed as observed species, tended to be higher in the gut of HIGH-ALPHA pigs (*p* = 0.051, Figure S7A, Supplementary Materials). Litter effect was the main factor influencing the microbiota relative to the diet effect considering either rare species or abundant species (R^2^ = 0.096, *p* = 0.002). Due to the diet effect, preterm pigs showed slightly different microbial composition, especially the rare species (R^2^ = 0.05, *p* = 0.028, Figure S7C, Supplementary Materials). Considering litter as a background effect, diet was the most important factor influencing the microbiota in contrast to sex (R^2^ = 0.065, *p* = 0.04, Figure S7D, Supplementary Materials). Figure S7E, Supplementary Materials shows the relative abundance of microbial genus composition after CSS normalization, among which 32 were annotated at genus level and 12 were unambiguously in upper levels. Species from *Lachnospiraceae*, *Clostridiaceae*, *Clostridiales* and *Clostridium* spp. were dominant genera in both HIGH-ALPHA and STANDARD-ALPHA, taking up nearly half of the total abundance. HIGH-ALPHA had a higher abundance of *Clostridiaceae*, *Enterobacteriaceae*, *Streptococcus* and *Streptomyces,* determined by ANCOM (Figure 5). No change was found at the class or phylum level. Results from correlation analyses between gut microbiome and structural and functional gut-, immunity- and brain-related outcomes showed no significant correlations among different microbial species (heat map data not presented).

### 3.7. Physical Activity, Behavior and Cognition

Acquisition of basic neuromuscular control, including time for eyelid opening, first stand, first walk and trough drinking was similar between HIGH-ALPHA and STANDARD-ALPHA (Figure S8, Supplementary Materials), and their physical activity during the first 10 days was the same (Figure S9A, Supplementary Materials), although STANDARD-ALPHA tended to show a higher number of activity bouts from Day 5–10 (*p* < 0.052, Figure S9B, Supplementary Materials). The distance travelled and velocity of the free movement behavior investigated at Day 9 in an open field did not differ between HIGH-ALPHA and STANDARD-ALPHA (data not shown), or between WPC and REF pigs (Table S2, Supplementary Materials). In the T-maze forward phases, the learning criterion of 80% correct choices was reached in the fourth acquisition for both WPC groups (Figure S9C, Supplementary Materials), with a similar proportion of pigs reaching the learning criterion at Acquisition 6 (88% HIGH-ALPHA and 93% STANDARD-ALPHA) (Figure S9D, Supplementary Materials). In the reverse phase (Acquisitions 7–9), the proportion of pigs reaching the learning criterion increased similarly from 0% for HIGH-ALPHA and STANDARD-ALPHA in the first reversal phase to 50% for both groups in the third and last reversal phase (Figure S9D, Supplementary Materials).

The WPC pigs required a shorter time for eyelid opening, first stand and first walk (*p* < 0.05, Table S2, Supplementary Materials), and they tended to have a higher activity level from Day 6–8 compared with REF (*p* = 0.061, Table S2, Supplementary Materials). Nevertheless, there were no differences between correct choice percentage in the T-maze between WPC and REF from Acquisition 1–9 (Table S2, Supplementary Materials).

## 4. Discussion

Nutrition is a critical factor for the neonatal growth of the preterm infant, and optimized infant formula with a protein and amino acid composition more similar to human milk is essential when human milk is not sufficient. In this study, we investigated if a WPC enriched with α-Lac improved growth as well as the structure and function of the gut, immunity and brain in preterm pigs as a model of preterm infants compared with a WPC containing a standard α-Lac concentration corresponding to human milk. Further, we investigated how the overall WPC fortification affected the same variables in comparison to a reference group without WPC. The WPC supplementation was well tolerated in both HIGH-ALPHA and STANDARD-ALPHA preterm pigs, with substantial body growth compared to the non-supplemented REF pigs. The WPC supplies the pigs with several whey proteins, including α-Lac. The good growth and gastrointestinal tolerability of preterm pigs receiving the WPC-supplemented diet is in accordance with α-Lac supplementation studies in infants [10,18,19]. Only minor effects of α-Lac-enrichment above human milk levels were observed compared to a WPC with α-Lac levels similar to human milk. Enrichment with α-Lac improved bone mineral density and relative bone mineral content relative to STANDARD-ALPHA. However, it must be taken into account that the bone mass of HIGH-ALPHA pigs represented a marginally larger part of the total body mass, since the relative weight gain was slightly lower than for STANDARD-ALPHA pigs. No additional benefits to brain cognitive outcomes were observed.

We observed similar growth of the majority of abdominal organ and gut parameters in the HIGH-ALPHA and STANDARD-ALPHA pigs indicated by similar relative intestinal weights, structural crypt and villi length and goblet cell density. Based on equivalent protein and energy intake from the WPC diets, the change in the WPC profile, with a 3-fold increase in α-Lac, did not change the overall metabolism in the preterm pigs, and the HIGH-ALPHA and STANDARD-ALPHA showed similar functional gut properties with regards to absorptive capacity and intestinal permeability. The WPC pigs had improved growth compared with REF for most abdominal organs and gut, as well as improved gut functional capacity, as indicated by a higher digestion of lactose and increased hexose absorption. The reduced intestinal growth in REF pigs, together with the low albumin and BUN levels and lower body weight gain, indicates the nutritional insufficiency of the diluted milk base diet, resulting in the mobilization of energy from abdominal organs to support growth. This is similar to many preterm infants, which is explained by a combination of inadequate nutrition and preterm-birth-related co-morbidities [51]. The undeveloped gut architecture, with lower intestinal crypt depths, probably explains the increased goblet cell density in REF, and the increased activity of sucrase, aminopeptidase A and dipeptidyl peptidase 4, may be a result of adaptation to the insufficient nutrition and a reduced absorptive and digestive capacity to optimize nutritional intake. The gut transit time was similar in HIGH-ALPHA and STANDARD-ALPHA throughout the study period, albeit with a tendency in HIGH-ALPHA pigs of a reduced gut transit time and numerically reduced gastric emptying time. Altogether, the chromium oxide tests throughout the study period may indicate a positive influence of α-Lac on gut motility and tolerance, similar to what has previously been observed [10].

Recent studies suggest that the gut microbiome directly affects the survival, growth and development of preterm infants with a long-term influence [52]. Appropriate milk proteins may diversify the gastrointestinal microbiome, similar to the effects of breast milk [22]. This would help avoid the dysbiosis seen in patients with NEC and/or sepsis [53]. Even though FISH analysis showed a similar bacterial abundance in the distal small intestine of HIGH-ALPHA and STANDARD-ALPHA, the minor increase in the number of different bacterial species in HIGH-ALPHA indicates a slight stimulation of beneficial bacteria with increased dietary α-Lac in the preterm gut. The differences were mainly found within phylogenetic similar species, as observed by the Shannon index. The gut microbiota of preterm infants is less diverse relative to full-term infants, and they are at greater risk of dysbiosis due to physiological and immune immaturity, along with external factors such as antibiotics treatment [52]. Antibiotics were also used in the current study, which may limit any influence of dietary treatments. Breast-fed infants have a colonic flora numerically dominated by *Bifidobacterium* strains, whilst formula-fed infants have a more complex microbiota composition, similar to adults [22,23]. Supplementation with additional α-Lac has been shown to have modulating prebiotic effects on the microflora by promoting the growth of certain bacterial groups in a manner similar to breast milk [23], especially *Bifidobacterium infantis* and *Bifidobacterium breve* [22,24]. In both HIGH-ALPHA and STANDARD-ALPHA pigs, strains from *Lachnospiraceae*, *Clostridiaceae*, *Clostridiales* and *Clostridium* dominated, but the HIGH-ALPHA pigs had a higher concentration of *Clostridiaceae*, *Enterobacteriaceae*, *Streptococcus* and *Streptomyces*. This is in accordance with findings in preterm infants, where the microbiome is characterized by high numbers of *Clostridiaceae*, *Streptococcaceae* and *Enterobacteriaceae* and very low numbers of *Bifidobacterium* and *Bacteroidetes* strains, in contrast with healthy term infants [54,55]. Likewise, the most abundant families in the REF pigs were found to be *Clostridiaceae*, *Enterobacteriaceae* and *Lachnospiraceae*, as analyzed in a previous study (Ahnfeldt, A.M.; Bæk, O. et al., Nutrient restriction has limited short-term effects on gut, immunity and brain development in preterm pigs. *J. Nutr.* (under review)). Results in preterm infants show that the initial colonization is less influenced by feeding type, since the most important determinant of microbiota composition is the degree of prematurity [54]. More pronounced effects of α-Lac might appear within a longer timeframe, when gut maturation is more profound.

There is growing evidence that the bidirectional communication within the microbiota–gut–brain axis is critical for health, cognition, brain and organ development [53,56]. The trajectories of microbiota-originated communication with organ systems and the brain might involve their metabolites, such as SCFAs [56]. The high lactose content in the WPC supplemented diets correlates well with the high levels of the lactose-digesting *Enterobacteriaceae* [57] found in WPC pigs and the corresponding high levels of lactate [55]. In contrast, *Streptococcaceae* and *Clostridiaceae* produces acetic acid from the fermentation of lactose [58,59]. Acetic acid is the principal SCFA, together with lactate, in breast-fed infants and also in infants fed α-Lac-supplemented milk [22,60]. Acetic- and butanoic acid was found in high concentrations in HIGH-ALPHA pigs compared with STANDARD-ALPHA, also relative to the levels of other microbial metabolites. Butanoic acid is produced by *Clostridium*, among others, utilized by the microbiota and the primary fuel for colonic epithelial cells [61]. Despite the lack of immediate neonatal cognitive effects in this study, previous studies show that butanoic acid plays an important role in the functioning of the microbiota–gut–brain axis [62,63] as well as in metabolic health [64]. Propanediol was also found in high concentrations in HIGH-ALPHA pigs. Propanediol is a precursor of propionate, a hepatic and intestinal gluconeogenic substrate [65] that may also correlate to lower NEC risk [66]. Together with the higher levels of several other microbial metabolites, this suggests an altered fermentation pattern after supplementation with high amounts of α-Lac, although no association between bacterial families/genera and microbial metabolites were found.

For immune parameters, total leukocytes, lymphocytes and immune cell counts, as well as the phagocytic function and capacity of neutrophils, were equal between HIGH-ALPHA and STANDARD-ALPHA, demonstrating a similar development of systemic immunity. Relative to REF at the corresponding days of life, neutrophil phagocytic function and capacity were much higher in WPC pigs, indicating a higher degree of immune maturation. Moreover, REF had higher circulating levels of several immune cell types, which was expected given the higher number of REF pigs with symptoms of ill health compared with WPC pigs (Ahnfeldt, A.M.; Bæk, O. et al., Nutrient restriction has limited short-term effects on gut, immunity and brain development in preterm pigs. *J. Nutr.* (under review)).

As regards hematological parameters, MPV was higher in HIGH-ALPHA compared to STANDARD-ALPHA, and WPC higher than REF, on Day 19. The MPV reflects the production and stimulation of platelets [67]. Correspondingly, REF had lower levels of circulating platelets. Larger platelets are more reactive and therefore shorten bleeding time [67]. Thus, the higher MPV, together with a higher bone mineral density and relative bone mineral content in HIGH-ALPHA, may be a sign of improved bone health [67] with the increased α-Lac-supplementation. The reduced levels of circulating monocytes, platelets and erythrocytes in REF relative to WPC pigs supports the indications of improved bone health and bone marrow production with WPC supplementation in general.

Only a few variations between HIGH-ALPHA and STANDARD-ALPHA were found in serum biochemical analyses, and their values were within the normal range, indicating no adverse effects of HIGH-ALPHA supplementation. The HIGH-ALPHA pigs did have higher levels of albumin, creatine kinase, creatinine, potassium and BUN than STANDARD-ALPHA pigs. Elevated creatinine and BUN values normally result from a high-protein diet [68], which was reflected in the higher BUN-values in WPC versus REF pigs. On Day 8, only the serum levels of calcium were reduced in the REF, but more pronounced differences developed over time and, on Day 19, the levels of several biochemical parameters (albumin, total protein, phosphate, BUN, calcium, magnesium and sodium) were reduced as well. Further, the liver weight was lower in REF. This indicates that the basic milk diet in the REF group was not sufficient to maintain a matching supply of nutrients and the renal and liver function may have been compromised compared with WPC.

Although α-Lac provides a high proportion of essential amino acids [10], the differences in the levels of α-Lac in the WPC products were not able to change the overall amino acid concentration in the full diets based on the dilute milk and the supplemented WPC with high and standard α-Lac levels, respectively. In plasma, four essential amino acids were higher in HIGH-ALPHA relative to STANDARD-ALPHA, including tryptophan, but were only transient on Day 8. Contrary to this, the WPC pigs had higher plasma levels for most of the amino acids. Human studies have shown that a diet composed of an α-Lac-enriched WPC increased the ratio of plasma tryptophan to other amino acids, and this prevented a cortisol response during acute experimental stress [69]. The uptake of the serotonin precursor [70] into the brain is dependent on nutrients that influence the cerebral availability of tryptophan via a raise in the ratio of plasma tryptophan to other large neutral amino acids [69,70]. However, neither serotonin nor cortisol were different between HIGH-ALPHA and STANDARD-ALPHA pigs on Day 19, but plasma cortisol levels were almost twice as high in REF relative to WPC pigs, indicating higher stress levels.

There were no differences in total brain weight, regional weight or cerebral water content in HIGH-ALPHA versus STANDARD-ALPHA, but, compared with REF, the weights of the hippocampus and striatum relative to total brain weight were higher in WPC pigs and there was a tendency for this in the brain stem as well, despite a higher cerebral water content being seen in the REF pigs. Cerebral water content decreases as the cerebral microstructure develops [71], and our results indicate that WPC supplementation affected cerebral microstructure development in the immediate neonatal period. REF required a longer time to acquire basic neuromuscular control, but the exploratory abilities of all pigs were tested in an open field at Day 9 and distance moved, as well as velocity of movement, were similar. Finally, results from the testing of the functional capacity for spatial orientation and memory in a T-maze system were similar between groups at Days 13–18. Thus, despite initial neurodevelopmental disabilities and differences in regional brain weights, the WPC supplementation did not improve exploratory abilities and cognition. Similar trends have been observed when comparing preterm pigs with term pigs, all fed bovine milk supplemented with colostrum. The preterm pigs displayed a delay in the acquisition of basic neuromuscular control but they caught up functionally with term pigs to reach the same moved distance and velocity as term pigs in the open field test [47]. In accordance with our functional results, studies have demonstrated the phenomenon of “brain sparring”, where the brain growth is partly independent of body growth during adverse conditions [72]. This could explain why the REF pigs have similar exploratory and cognitive abilities despite a lower body growth, and development of orientation, memory and cognition in preterms may be relatively unaffected by diet in the immediate neonatal period.

To meet the amino acid requirements of infants, especially regarding tryptophan and cysteine, most bovine milk-based formulas contain more protein (13–15 g/L) than human milk (9.5 g/L) [73]. However, excessive protein intake can induce metabolic stress on immature kidneys and liver, as well as disproportionate weight gain and metabolic programming, resulting in an increased risk of obesity later in life [73]. With the optimal amino acid composition of α-Lac, several studies have shown that the total protein concentration can be lowered in formula if the concentration of α-Lac is increased [10,18,19]. In this study, we tested the effects of increasing amounts of α-Lac on growth and functional organ development. STANDARD-ALPHA pigs received a milk diet with an α-Lac concentration slightly higher than what is found in human transitional milk (6.3 versus 3.5 g/L, respectively), whereas the HIGH-ALPHA pigs received a milk diet with 18 g/L α-Lac. This corresponds to a daily dose of 0.96–1.1 g and 2.7–3.2 g α-Lac per kg body weight per day in STANDARD-ALPHA and HIGH-ALPHA, respectively. With an average daily milk intake in breastfed infants of approximately 750 mL milk per day during the first four months of life [74], this corresponds to an approximate intake of 0.5–0.8 g α-Lac per kg body weight per day in healthy newborn infants [75]. The daily doses tested in the preterm pigs (0.96–1.1 g α-Lac per kg body weight per day in STANDARD-ALPHA and 2.7–3.2 g α-Lac per kg body weight per day in HIGH-ALPHA) were therefore similar to, and about 3-fold higher, respectively, than what is normally ingested in infants, and we have not tested if lower levels of α-Lac compromise growth and organ development in the newborn. From the results obtained, it appears that, although the WPCs were well tolerated, the provision of additional α-Lac to infant formula, beyond the levels found in human milk, does not provide additional benefits to the sensitive newborns in terms of improved cognitive outcome within the immediate neonatal period. Minor effects on body composition and gut microbiota did, however, appear. Whether cognitive outcomes would be improved within a longer time period is unknown, and we cannot exclude the possibility that the changes in gut microbiota may have indirect effects on the gut, immunity and brain after a longer time period.

We conclude that feeding preterm pigs bovine milk supplemented with a WPC enriched with α-Lac in the immediate neonatal period slightly altered the composition of the body and gut microbiota, but α-Lac enrichment beyond the levels found in human milk did not change overall gut, immunity and brain function as well as cognition compared with a WPC with a level of α-Lac similar to human milk. Relative to preterm pigs fed a nutrient-restricted bovine milk diet, WPC-supplementation resulted in pronounced beneficial effects on the growth and functional development of body and organ systems, gut, immunity and structural brain development, although specific α-Lac enrichment of WPC above human milk levels failed to provide further benefits in this study.

## Figures and Tables

**Table 1:** Nutrient content in milk diets supplemented with bovine WPC enriched with α-Lac (HIGH-ALPHA), with standard α-Lac content (STANDARD-ALPHA) or without WPC supplementation (REF) for preterm pigs.

Nutrient Content	HIGH-ALPHA	STANDARD-ALPHA	REF
Energy (kJ/L)	2902	3012	2426
Protein (g/L)	55.0	55.0	27.0
Carbohydrate (g/L)	33.0	36.0	33.0
Fat (g/L)	38.0	40.0	38.0
α-lactalbumin (g/L)	18.0	6.3	0.7
Osmolality (mOsm/kg)	200	222	190

**Table 2:** Organ weights in 19 day old preterm pigs fed bovine milk diets supplemented with WPC with high (HIGH-ALPHA) or standard α-Lac content (STANDARD-ALPHA) (mean ± SD, *n* = 17–18 in each group).

Absolute Weight (g)				Relative Weight (g/kg)		
Organ	HIGH-ALPHA	STANDARD-ALPHA	*p*	HIGH-ALPHA	STANDARD-ALPHA	*p*
Small intestine	56.1 ± 14	65.7 ± 19	ns	37.8 ± 5.0	41.5 ± 6.5	*
Small intestinal length (cm)	417 ± 41	461 ± 63	**	-	-	ns
Proximal small intestine	19.9 ± 5.6	23.6 ± 7.0	**	13.3 ± 1.5	15.0 ± 2.7	*
Mid small intestine	17.4 ± 4.4	20.3 ± 7.1	*	11.8 ± 2.2	12.7 ± 2.6	ns
Distal small intestine	18.8 ± 5.0	21.8 ± 6.1	*	12.7 ± 1.8	13.8 ± 2.3	ns
Stomach	9.21 ± 2.5	9.89 ± 4.0	ns	6.16 ± 0.7	6.26 ± 1.9	ns
Colon	40.5 ± 20	38.7 ± 19	ns	25.4 ± 12	25.4 ± 14	ns
Liver	40.3 ± 12	40.1 ± 12	ns	26.8 ± 3.3	25.2 ± 6.0	ns
Spleen	5.47 ± 2.3	5.83 ± 2.0	ns	3.60 ± 1.0	3.39 ± 1.1	ns
Heart	10.2 ± 3.0	10.8 ± 2.8	ns	6.79 ± 0.9	6.89 ± 1.0	ns
Lungs	34.6 ± 11	33.5 ± 10	ns	23.3 ± 4.6	20.2 ± 7.8	ns
Kidneys	10.5 ± 2.3	11.9 ± 2.2	0.08	7.10 ± 0.9	7.65 ± 1.2	ns
Adrenal glands	0.37 ± 0.1	0.33 ± 0.1	ns	0.25 ± 0.1	0.21 ± 0.1	0.08

Relative values correspond to weight of organ relative to body weight. *p* = 0.08 indicates a tendency to a difference between groups. * *p* < 0.05; ** *p* < 0.01; ns: not significant.

## Supplementary Materials

The following are available online at https://www.mdpi.com/2072-6643/12/1/245/s1, Figure S1: Growth in preterm pigs, Figure S2: Dual-energy X-ray absorptiometry (DEXA) scans of 19 d-old preterm pigs, Figure S3: Milk and plasma concentrations of amino acids in preterm pigs, Figure S4: Phagocytic activity, Figure S5: Structural and functional gut endpoints measured in 5 and 19 day old preterm pigs, Figure S6: Gastric emptying and gut transit time measured in preterm pigs, Figure S7: Gut microbiota determined by the 16s rRNA gene amplicon sequencing at Day 19 in preterm pigs, Figure S8: Acquisition of basic motor skills and learning ability during the first eight days of life in preterm pigs, Figure S9: Physical activity in home cages from Days 1–10 of life for preterm pigs, Table S1: Brain weights and regional proportions in 19 day old preterm pigs fed bovine milk diets supplemented with WPC with high (HIGH-ALPHA) or standard α-Lac content (STANDARD-ALPHA) (mean ± SD, *n* = 17–18 in each group), Table S2: Structural and functional endpoints for preterm pigs fed a bovine milk diet, WPC supplemented (WPC) and reference pigs (REF). Parameters for proportional organ and brain regional weights, biochemistry, amino acids, hematology, immune cell counts, lymphocyte subsets, phagocytosis, gut endpoints, hexose absorption, microbial metabolite concentrations in plasma, and functional neurodevelopmental and cognitive results, Table S3: Biochemical parameters in serum at Days 8 and 19 in preterm pigs fed bovine milk diets supplemented with WPC with high (HIGH-ALPHA) or standard α-Lac content (STANDARD-ALPHA) (mean ± SD, *n* = 17–18 in each group), Table S4: Hematology, lymphocyte subsets and phagocytosis analyzed in blood collected at birth, at Days 8 and 19 in preterm pigs fed a bovine milk diet supplemented with WPC with high (HIGH-ALPHA) or standard α-Lac content (STANDARD-ALPHA) (mean ± SD, *n* = 19 in each group).
