# Supplementary material for: Alpha-Lactalbumin Enriched Whey Protein Concentrate to Improve Gut, Immunity and Brain Development in Preterm Pigs
Source: Nutrients. 2020 Jan 17;12(1):245. doi: 10.3390/nu12010245 (PMC7020014; doi:10.3390/nu12010245)
Supplement: Supplementary file 1 [file nutrients-12-00245-s001.pdf]

Supplementary Materials

Figure S1

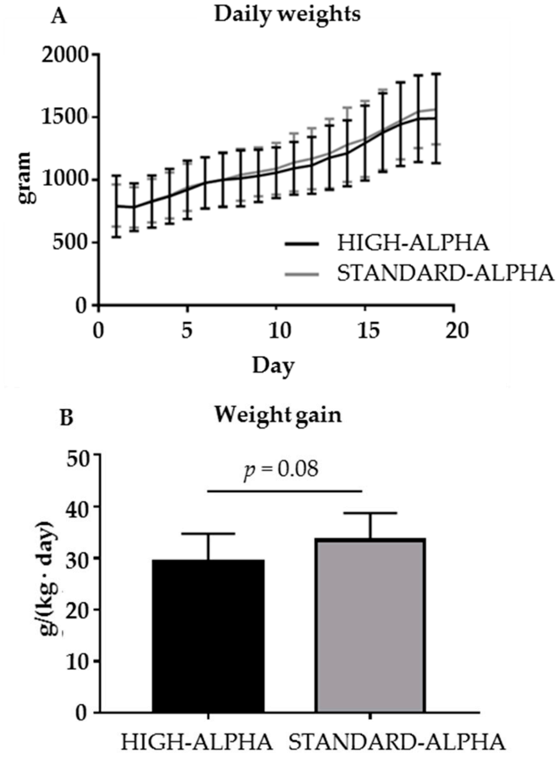

Figure S1. Growth in preterm pigs. (**A**) Daily body weights from Day 1-19 of preterm pigs fed a bovine milk diet supplemented with bovine whey protein concentrate either enriched with α-Lac (HIGH-ALPHA, n = 18) or with standard content of α-Lac (STANDARD-ALPHA, n = 17) and (**B**) Daily weight gain relative to body weight from Day 1-19. Data are expressed as mean ± SD. p = 0.08 indicates a tendency to a difference between groups.

Figure S2

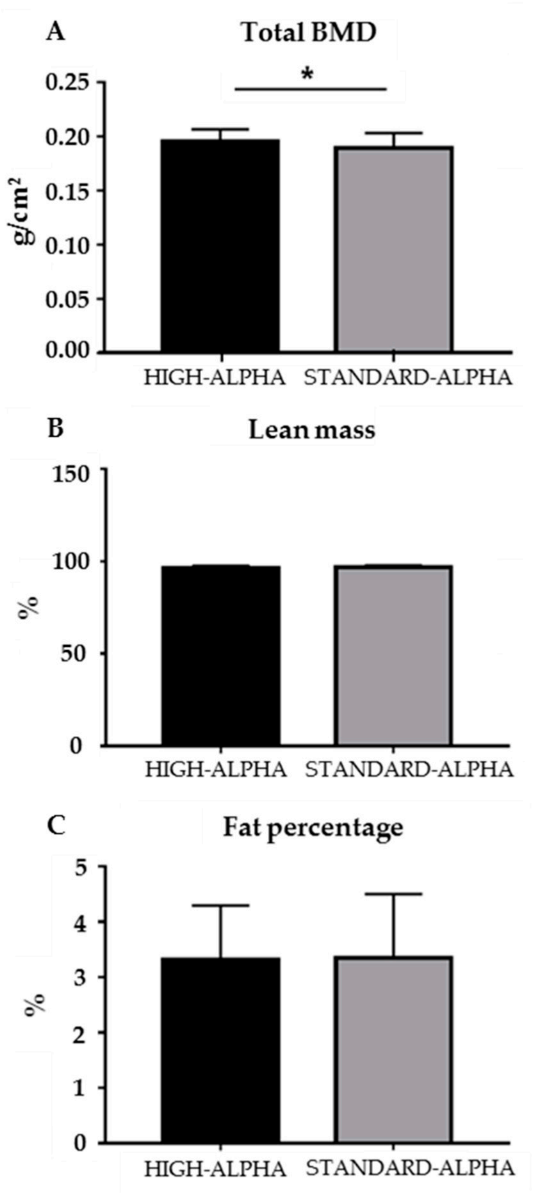

Figure S2. Dual-energy X-ray absorptiometry (DEXA) scans of 19 day old preterm pigs fed a bovine milk diet supplemented with bovine whey protein concentrate either enriched with α-Lac (HIGH-ALPHA, n = 16) or with standard content of α-Lac (STANDARD-ALPHA, n = 17). (**A**) Total bone mineral density (BMD); (**B**) Lean mass percentage and (**C**) Fat percentage. Data are expressed as mean ± SD. Significant differences between groups are shown (* $p < 0.05$).

Figure S3

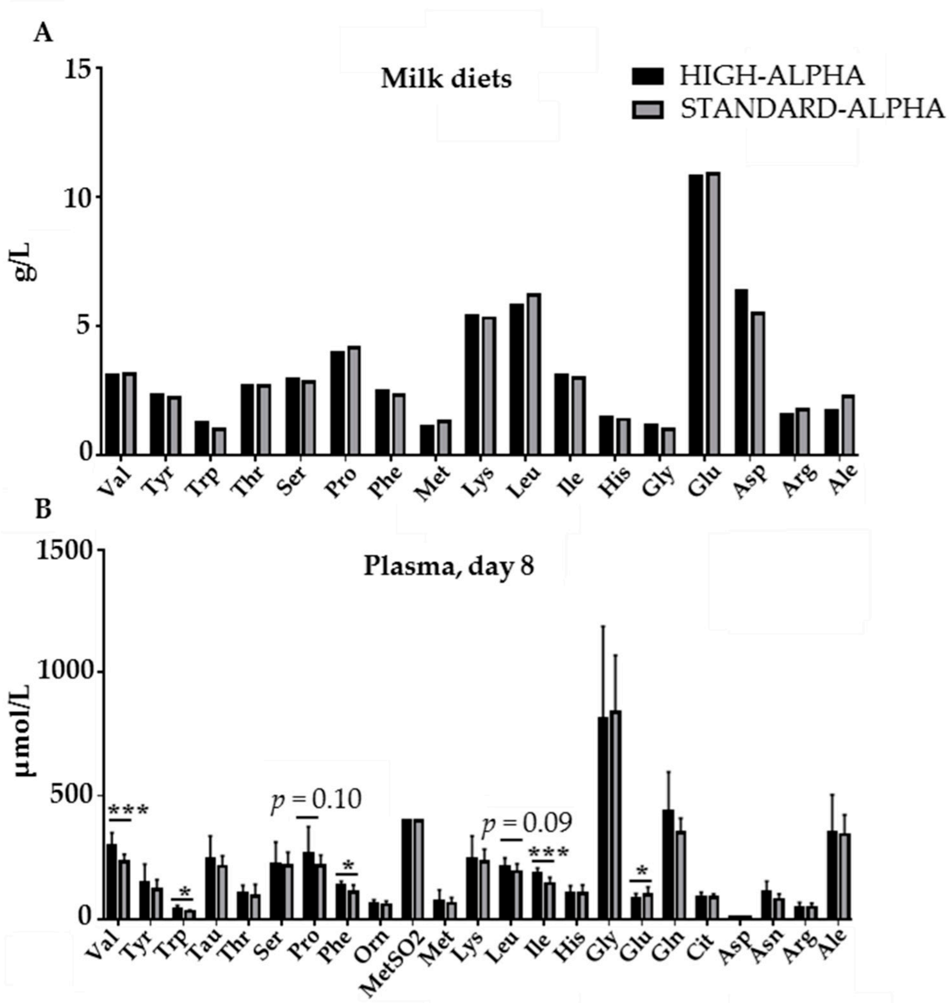

Figure S3. Milk and plasma concentrations of amino acids in preterm pigs fed a bovine milk diet supplemented with bovine whey protein concentrate either enriched with α-Lac (HIGH-ALPHA, n = 15) or with standard content of α-Lac (STANDARD-ALPHA, n = 15). (**A**) Measured in both milk diets and (**B**) in blood samples at Day 8. Tau, taurine; Orn, orhnithine; MetSO2, methionine sulfoxide; Gln, glutamine; Cit, citrulline and Asn, asparagine not measured in milk. Data are expressed as mean ± SD. p = 0.09 and 0.10 indicate tendencies to a difference between groups. Significant differences between groups are shown (* $p < 0.05$; *** $p < 0.001$).

Figure S4

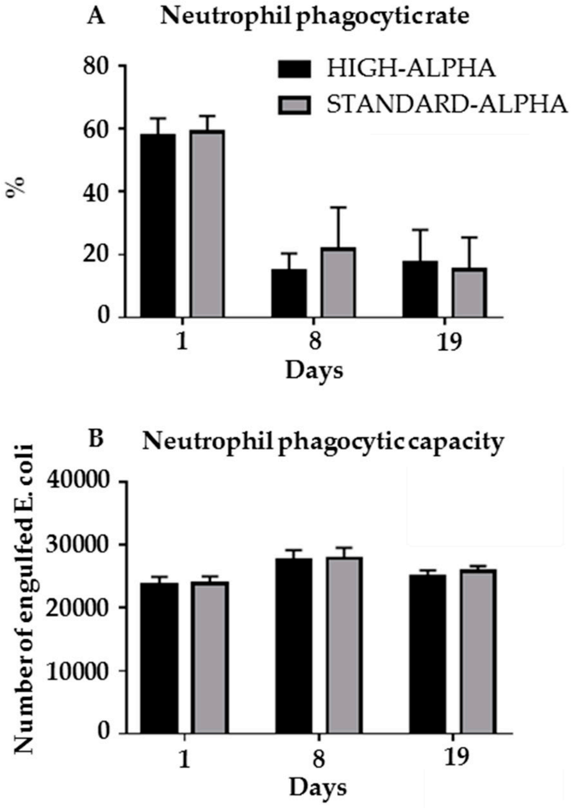

Figure S4. Phagocytic activity. (**A**) Phagocytic rate and (**B**) phagocytic capacity of neutrophils measured in blood samples after birth and in 8 and 19 day old preterm pigs fed a bovine milk diet supplemented with bovine whey protein concentrate either enriched with α-Lac (HIGH-ALPHA, n = 15) or with standard content of α-Lac (STANDARD-ALPHA, n = 20). Data are expressed as mean ± SD.

Figure S5

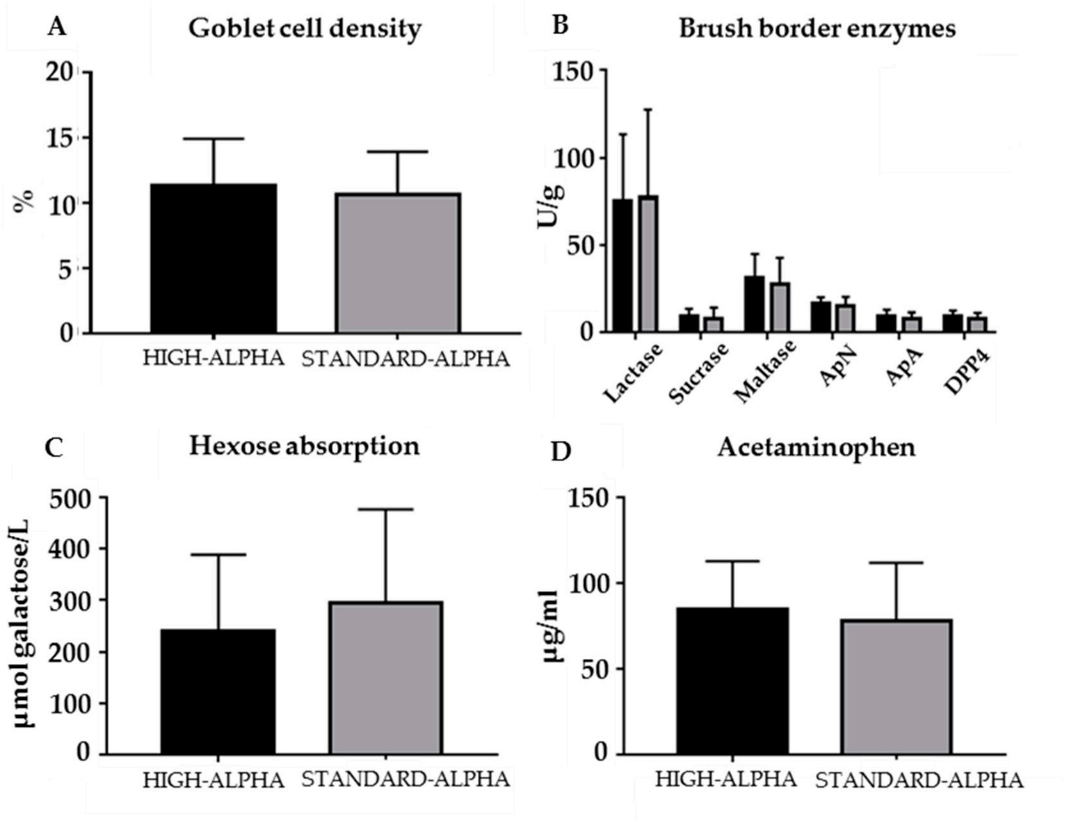

Figure S5. Structural and functional gut endpoints measured in 5 and 19 day old preterm pigs fed a bovine milk diet supplemented with bovine whey protein concentrate either enriched with α-Lac (HIGH-ALPHA, n = 15-18) or with standard content of α-Lac (STANDARD-ALPHA, n = 13-17). (**A**) Goblet cell density; (**B**) Brush border enzymes (lactase; sucrase; maltase; ApN, aminopeptidase N; ApA, aminopeptidase A; DPP4, dipeptidyl peptidase 4); (**C**) absorptive capacity of hexose and (**D**) gastric emptying of acetaminophen. Data are expressed as mean ± SD.

Figure S6

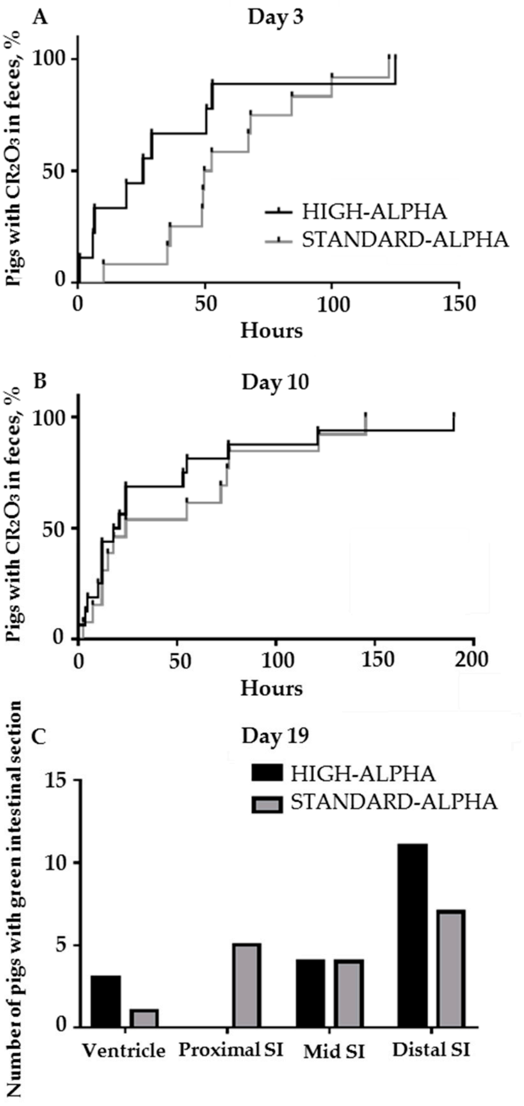

Figure S6. Gastric emptying and gut transit time measured in preterm pigs fed a bovine milk diet supplemented with bovine whey protein concentrate either enriched with α-Lac (HIGH-ALPHA, n = 18) or with standard content of α-Lac (STANDARD-ALPHA, n = 17). (**A**) Hours from oral ingestion of chromium oxide (CR_2O_3) until green-colored feces at Day 3 and (**B**) at Day 10. (**C**) Number of pigs with distal accumulation of chromium oxide (CR_2O_3) observed by green-colored intestinal section one hour after oral ingestion at Day 19 (p = 0.07 indicating a tendency to a difference between groups). Data are expressed as mean ± SD.

Figure S7

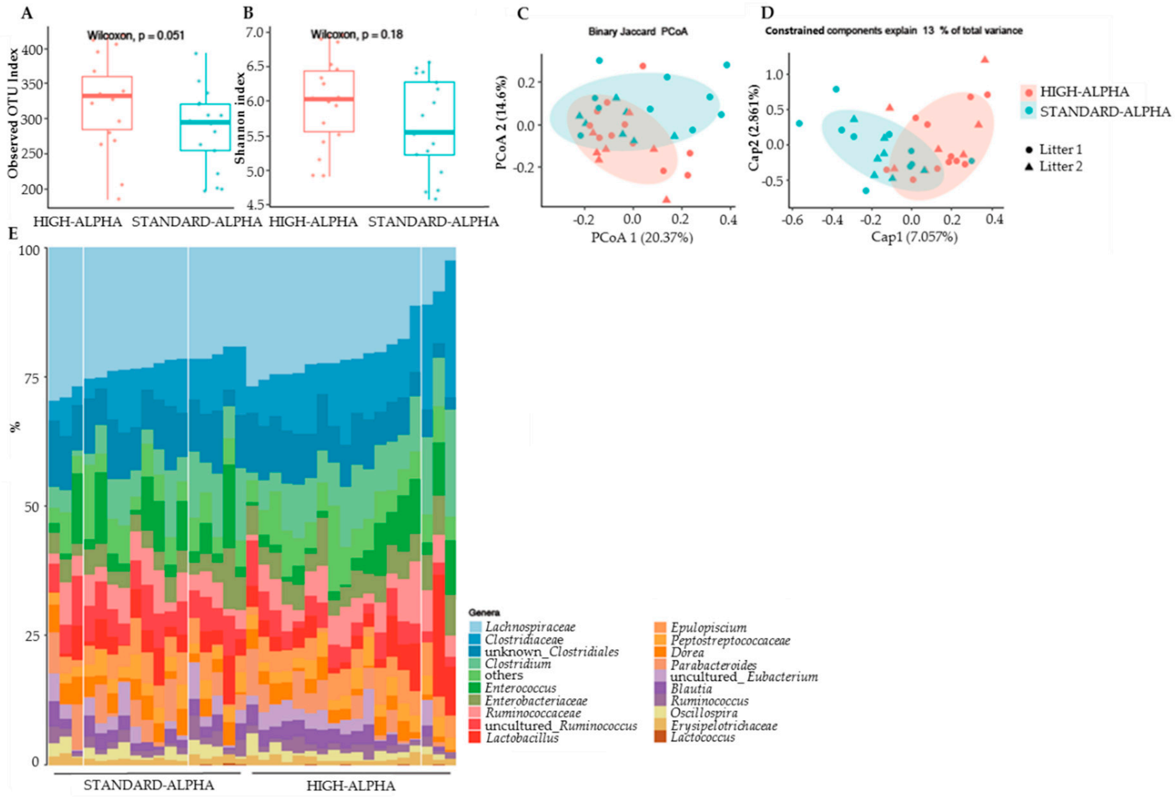

Figure S7. Gut microbiota determined by the 16s rRNA gene amplicon sequencing at Day 19 in preterm pigs fed a bovine milk diet supplemented with bovine whey protein concentrate either enriched with α-Lac (HIGH-ALPHA, $n = 18$) or with standard content of α-Lac (STANDARD-ALPHA, $n = 17$). (**A**) Total observed zOTU, zero-radius Operational Taxonomic Units numbers; (**B**) Shannon index; (**C**) Principal Coordinates analysis (PCoA) plot on the unweighted UniFrac distance matrix and (**D**) constrained analysis. Respective ellipses are drawn at 80% confidence level following multivariate t-distribution. (**E**) Relative abundance of prokaryotic gut microbiota members at genus level. Genera with a mean relative abundance below 1% were included in others and taxonomy annotations are italicized for genus and species level.

Figure S8

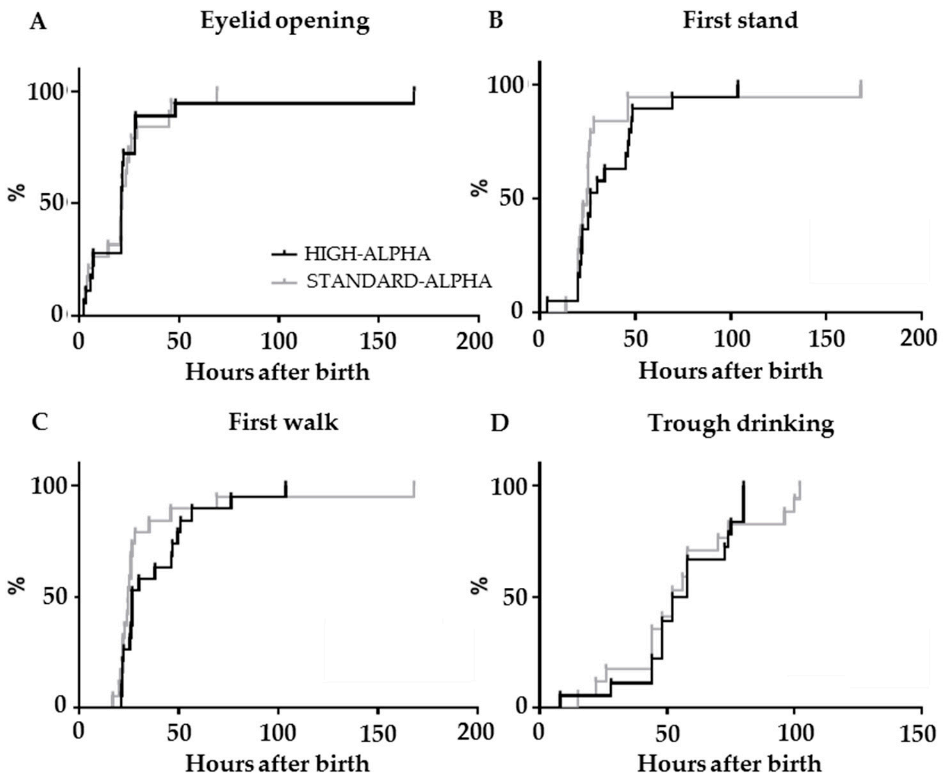

Figure S8. Acquisition of basic motor skills and learning ability during the first 8 days of life in preterm pigs fed a bovine milk diet supplemented with bovine whey protein concentrate either enriched with α-Lac (HIGH-ALPHA, $n = 18$) or with standard content of α-Lac (STANDARD-ALPHA, $n = 17$). (**A**) The proportion of pigs that were able to first open their eyes; (**B**) stand or (**C**) walk and (**D**) to learn independent drinking, beginning from Day 6 of life. Data are expressed as mean ± SD.

Figure S9

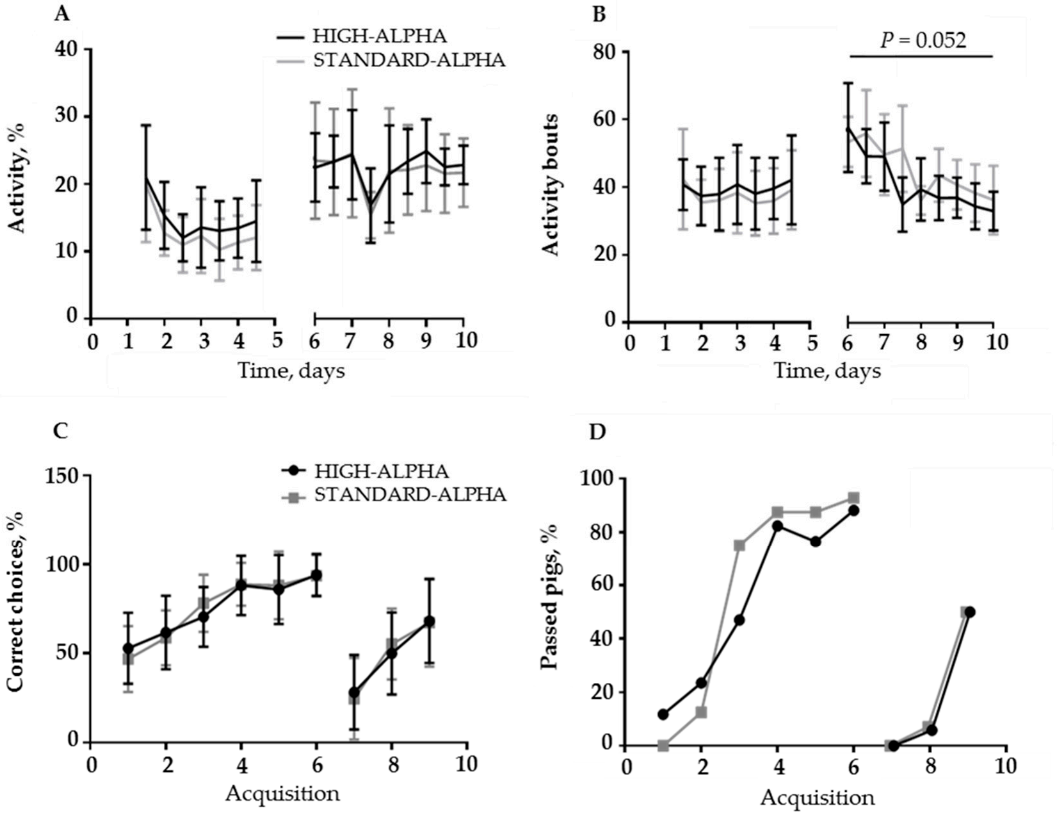

Figure S9. Physical activity in home cages from Day 1-10 of life for preterm pigs fed a bovine milk diet supplemented with bovine whey protein concentrate either enriched with α-Lac (HIGH-ALPHA, n = 18) or with standard content of α-Lac (STANDARD-ALPHA, n = 17). (**A**) Proportion active time and (**B**) number of active periods lasting more than 5 seconds, expressed as activity bouts. (**C**) T-maze performance from Day 13-18 of life. Correct choices in forward phases in Acquisition 1-6 and reverse phases in Acquisition 7-9. (**D**) Proportion passed pigs reaching learning criteria of 80% correct choices in one acquisition. Data are expressed as mean ± SD. p = 0.052 indicates a tendency to a difference between groups.

Table S1

Table S1. Brain weights and regional proportions in 19 day old preterm pigs fed bovine milk diets supplemented with WPC with high (HIGH-ALPHA) or standard α-Lac content (STANDARD-ALPHA) (mean ± SD, n = 17-18 in each group).

	**Absolute weight (g)**			**Relative weight (g/kg)**		
	**HIGH-ALPHA**	**STANDARD-ALPHA**	***p***	**HIGH-ALPHA**	**STANDARD-ALPHA**	***p***
Total brain	34.1 ± 3.1	34.3 ± 2.5	ns	2.38 ± 0.43	2.24 ± 0.37	ns
Cerebrum	27.2 ± 2.6	27.2 ± 2.3	ns	79.7 ± 1.1	79.4 ± 1.3	ns
Cerebellum	3.70 ± 0.3	3.71 ± 0.3	ns	10.9 ± 0.6	10.8 ± 0.7	ns
Hippocampus	0.57 ± 0.1	0.63 ± 0.2	ns	1.69 ± 0.2	1.83 ± 0.4	ns
Brain stem	3.13 ± 0.3	3.15 ± 0.3	ns	9.19 ± 0.4	9.21 ± 0.6	ns
Striatum	0.44 ± 0.1	0.46 ± 0.1	ns	1.30 ± 0.3	1.34 ± 0.2	ns
Wet weight remnant	11.6 ± 1.2	11.6 ± 1.0	ns	-	-	ns
Dry weight remnant	2.02 ± 0.2	2.04 ± 0.2	ns	-	-	ns
Cerebral water content	82.5 ± 0.3	82.5 ± 0.5	ns	-	-	ns

Relative values correspond to weight of brain region relative to total brain weight. ns: not significant.

Table S2

Table S2. Structural and functional endpoints for preterm pigs fed a bovine milk diet WPC supplemented (WPC) and reference pigs (REF). Parameters for proportional organ and brain regional weights, biochemistry, amino acids, hematology, immune cell counts, lymphocyte subsets, phagocytosis, gut endpoints, hexose absorption, microbial metabolite concentrations in plasma, and functional neurodevelopmental and cognitive results. All endpoints were analyzed at birth, at Day 8 or 19 in preterm pigs (mean ± SD; WPC (pooled values of HIGH-ALPHA and STANDARD-ALPHA), *n* = 35; REF, *n* = 18 pigs).

	Day	WPC	REF	*p*
**Organ weights**				
Small intestine (g/kg)	19	39.6 ± 6.00	31.0 ± 8.50	***
Small intestinal length (cm)	19	438 ± 56.34	402 ± 44.72	***
Proximal small intestine (g/kg)	19	14.10 ± 2.31	11.38 ± 1.55	***
Mid small intestine (g/kg)	19	12.27 ± 2.43	10.20 ± 1.62	**
Distal small intestine (g/kg)	19	13.22 ± 2.13	10.79 ± 3.32	**
Stomach (g/kg)	19	6.21 ± 1.38	6.41 ± 0.99	ns
Colon (g/kg)	19	25.41 ± 12.52	13.67 ± 9.54	**
Liver (g/kg)	19	26.06 ± 4.75	21.49 ± 2.34	***
Spleen (g/kg)	19	3.50 ± 1.04	2.26 ± 1.09	***
Heart (g/kg)	19	6.84 ± 0.92	7.58 ± 1.44	*
Lungs (g/kg)	19	21.78 ± 6.46	20.92 ± 8.27	ns
Kidneys (g/kg)	19	7.37 ± 1.07	6.92 ± 1.01	ns
Adrenals (g/kg)	19	0.23 ± 0.07	0.22 ± 0.10	ns
**Brain regional weights**				
Total brain (%)	19	2.31 ± 0.40	2.33 ± 0.43	ns
Cerebrum (%)	19	79.50 ± 1.20	80.24 ± 1.32	0.058
Cerebellum (%)	19	10.84 ± 0.67	10.87 ± 1.09	ns
Hippocampus (%)	19	1.76 ± 0.31	1.51 ± 0.24	**
Brain stem (%)	19	9.20 ± 0.49	8.89 ± 0.59	0.059
Striatum (%)	19	1.32 ± 0.25	1.00 ± 0.21	***
Wet weight remnant	19	11.60 ± 1.12	12.06 ± 1.30	ns
Dry weight remnant	19	2.03 ± 0.18	2.06 ± 0.28	ns
Cerebral water content	19	82.50 ± 0.42	82.93 ± 0.90	*
**Biochemistry**				
Albumin (g/L)	8	12.12 ± 1.75	11.72 ± 2.72	ns
	19	20.26 ± 3.49	14.52 ± 4.83	***
Total protein (g/L)	8	27.09 ± 2.99	27.22 ± 5.78	ns
	19	33.73 ± 6.38	25.47 ± 7.62	**
BASP (U/L)	8	1247 ± 442	1520 ± 625	0.099
	19	1177 ± 344	1794 ± 850	**
ALAT (U/L)	8	22.96 ± 7.38	23.40 ± 13.09	ns
	19	42.00 ± 8.26	37.20 ± 10.24	ns
Total bilirubin (µmol/L)	8	5.48 ± 2.26	4.73 ± 2.84	ns
	19	1.00 ± 0.94	0.08 ± 0.63	ns
Cholesterol (mmol/L)	8	2.72 ± 0.48	3.07 ± 1.77	ns
	19	2.91 ± 0.59	3.49 ± 0.90	*
Creatinine (umol/L)	8	47.93 ± 6.92	59.47 ± 20.31	**
	19	47.25 ± 9.13	47.30 ± 17.39	ns
Creatine kinase (U/L)	8	52.37 ± 57.24	82.93 ± 56.29	0.058
	19	414 ± 531	126 ± 58.22	ns
Iron (µmol/L)	8	4.79 ± 3.00	9.76 ± 6.94	**
	19	6.48 ± 5.55	11.79 ± 3.04	**
Phosphate (mmol/L)	8	1.99 ± 0.21	2.15 ± 0.41	0.051
	19	2.59 ± 0.51	2.15 ± 0.58	*
AST (U/L)	8	27.26 ± 12.54	24.33 ± 11.69	ns
	19	48.18 ± 28.59	31.00 ± 10.25	0.076

BUN (mmol/L)	8	2.22 ± 1.64	2.03 ± 2.39	ns
	19	7.64 ± 2.64	2.00 ± 1.11	***
GGT (U/L)	8	18.04 ± 6.71	25.53 ± 10.62	*
	19	19.14 ± 6.51	21.80 ± 8.61	ns
Calcium (mmol/L)	8	2.71 ± 0.15	2.49 ± 0.38	**
	19	3.04 ± 0.42	2.26 ± 0.54	***
Magnesium (mmol/L)	8	0.83 ± 0.11	0.86 ± 0.29	ns
	19	1.08 ± 0.21	0.91 ± 0.24	*
Sodium (mmol/L)	8	148 ± 5.47	149 ± 18.44	ns
	19	152 ± 21.98	129 ± 27.11	*
Potassium (mmol/L)	8	4.79 ± 0.66	5.53 ± 1.09	*
	19	5.81 ± 0.77	5.43 ± 1.28	ns
Lactate (mmol/L)	8	2.67 ± 2.11	3.8 ± 2.7	ns
	19	8.80 ± 3.25	8.33 ± 2.73	ns
Glucose (mmol/L)	8	3.30 ± 0.80	3.15 ± 1.52	ns
	19	5.01 ± 1.17	5.48 ± 1.61	ns
Insulin[A] (mU/L)	19	2.63 ± 1.28	2.39 ± 0.27	ns
GLP-1[A] (pmol/L)	19	9.74 ± 12.13	10.50 ± 11.92	ns
Cortisol (ng/ml)	19	38.10 ± 55.61	70.40 ± 106	**
**Amino acids**				
Valine (μmol/L)	8	266 ± 53.89	273 ± 74.42	ns
	19	419 ± 147	223 ± 113.57	***
Tyrosine (μmol/L)	8	134 ± 60.58	158 ± 196	ns
	19	197 ± 76.33	145 ± 82.44	0.086
Tryptophan (μmol/L)	8	35.95 ± 11.93	16.32 ± 3.76	***
	19	46.61 ± 16.66	17.33 ± 9.89	***
Threonine (μmol/L)	8	229 ± 72.94	138 ± 78.12	***
	19	405 ± 39.01	164 ± 43.04	***
Taurine (μmol/L)	8	99.65 ±39.48	55.96 ±27.93	***
	19	166 ± 39.01	50.18 ± 18.50	***
Serine (μmol/L)	8	220 ± 71.33	203 ± 64.14	ns
	19	213 ± 74.61	126 ± 41.30	**
Proline (μmol/L)	8	342 ± 84.74	264 ± 80.58	ns
	19	488 ± 169	286 ± 118	**
Phenylalanine (μmol/L)	8	122 ±26.89	102 ± 31.85	*
	19	131 ± 39.69	77.22 ± 33.02	***
Ornithine (μmol/L)	8	59.96 ± 15.36	42.52 ± 23.28	**
	19	90.28 ± 30.36	58.64 ± 34.92	*
Methionine (μmol/L)	8	69.45 ± 34.77	55.29 ± 19.76	0.094
	19	102 ± 84.19	95.73 ± 137	ns
Lysine (μmol/L)	8	238 ± 73.34	126 ± 57.62	***
	19	317 ± 132	169 ± 54.74	0.058
Leucine (μmol/L)	8	202 ± 35.13	106 ± 57.62	***
	19	308 ± 94.94	111 ± 62.27	***
Isoleucine (μmol/L)	8	164 ± 29.97	89.80 ± 30.02	***
	19	233 ± 68.31	126 ± 76.05	***
Histidine (μmol/L)	8	102 ± 33.37	163 ± 87.47	**
	19	99.21 ± 30.06	71.61 ± 26.61	*
Glycine (μmol/L)	8	827 ± 303	949 ± 391	ns
	19	677 ± 268	579 ± 283	ns
Glutamine (μmol/L)	8	393 ± 127	335 ± 125	**
	19	489 ± 118	390 ± 133	ns
Glutamic acid (μmol/L)	8	90.15 ± 26.73	93.41 ± 36.39	ns
	19	45.43 ± 16.79	52.43 ± 15.16	ns
Citric acid (μmol/L)	8	88.40 ± 17.10	129 ± 43.40	***
	19	134 ± 39.66	129 ± 41.16	ns
Aspartic acid (μmol/L)	8	8.20 ± 2.10	10.80 ± 4.02	**
	19	7.13 ± 2.19	10.82 ± 3.75	***

Asparagine (µmol/L)	8	95.84 ± 36.79	52.85 ± 20.07	**
	19	155 ± 55.47	56.48 ± 19.81	***
Arginine (µmol/L)	8	46.76 ± 18.80	30.83 ± 14.70	**
	19	50.43 ± 30.02	29.63 ± 10.77	*
Alanine (µmol/L)	8	347 ± 119	456 ± 148	*
	19	470 ± 136	337 ± 124	*
**Hematology**				
Erythrocytes (10^{12} cells/L)	1	3.98 ± 0.36	3.62 ± 0.26	***
	8	3.71 ± 0.51	3.71 ± 0.84	ns
	19	4.25 ± 0.56	4.33 ± 0.49	ns
Hemoglobin (mmol/L)	1	5.30 ± 0.40	5.12 ± 0.34	ns
	8	4.48 ± 0.51	4.66 ± 1.03	ns
	19	4.39 ± 0.92	4.82 ± 0.65	ns
Hematocrit (%)	1	0.38 ± 0.44	0.30 ± 0.02	ns
	8	0.33 ± 0.41	0.26 ± 0.06	ns
	19	0.26 ± 0.37	0.27 ± 0.03	ns
MCV (fL)	1	76.25 ± 4.28	80.46 ± 7.13	**
	8	67.55 ± 2.64	69.52 ± 2.96	*
	19	62.41 ± 3.63	62.11 ± 2.49	ns
MCHC (mmol/L)	1	17.55 ± 0.70	17.28 ± 0.49	ns
	8	17.70 ± 0.48	18.07 ± 0.46	*
	19	16.55 ± 2.66	17.89 ± 0.30	ns
Platelets (10^9 cells/L)	1	305 ± 38.01	244 ± 85.97	**
	8	448 ± 149	442 ± 312	ns
	19	673 ± 220	548 ± 116	0.097
MPV (fL)	1	10.74 ± 1.21	11.23 ± 1.22	ns
	8	12.69 ± 3.88	14.38 ± 5.93	ns
	19	9.54 ± 3.66	8.91 ± 0.83	*
MPC (g/L)	1	230 ± 6.19	233 ± 7.02	ns
	8	244 ± 7.50	232 ± 8.97	***
	19	240 ± 8.74	240 ± 7.11	ns
**Immune cell counts**				
Leukocytes (10^9 cells/L)	1	2.61 ± 0.41	2.82 ± 0.59	ns
	8	6.36 ± 2.40	7.60 ± 4.11	ns
	19	9.37 ± 3.66	12.50 ± 4.06	*
Neutrophils (10^9 cells/L)	1	0.57 ± 0.16	0.71 ± 0.30	*
	8	4.82 ± 3.24	4.95 ± 3.34	ns
	19	5.19 ± 3.04	8.80 ± 3.95	**
Neutrophils (%)	1	21.89 ± 5.55	24.75 ± 9.11	ns
	8	69.52 ± 11.91	64.73 ± 15.93	ns
	19	52.55 ± 14.18	67.72 ± 13.23	**
Lymphocytes (10^9 cells/L)	1	1.96 ± 0.37	2.00 ± 0.51	ns
	8	1.55 ± 0.65	2.32 ± 1.89	0.056
	19	3.13 ± 0.93	3.18 ± 1.16	ns
Lymphocytes (%)	1	75.07 ± 6.09	71.17 ± 9.96	0.086
	8	26.36 ± 11.29	30.53 ± 15.98	ns
	19	38.65 ± 13.24	27.79 ± 12.60	0.053
Monocytes (10^9 cells/L)	1	0.04 ± 0.02	0.05 ± 0.0	ns
	8	0.14 ± 0.13	0.16 ± 0.10	ns
	19	0.50 ± 0.42	0.25 ± 0.06	*
Monocytes (%)	1	1.59 ± 0.67	1.71 ± 1.16	ns
	8	2.15 ± 1.56	2.32 ± 1.43	ns
	19	5.52 ± 3.75	2.20 ± 0.83	**
Eosinophils (10^9 cells/L)	1	0.02 ± 0.03	0.05 ± 0.04	**
	8	0.03 ± 0.02	0.04 ± 0.05	*
	19	0.04 ± 0.11	0.08 ± 0.08	ns
Eosinophils (%)	1	0.78 ± 0.97	1.77 ± 1.44	**
	8	0.35 ± 0.22	0.76 ± 0.86	*

	19	0.45 ± 0.82	0.58 ± 0.64	ns
	1	0.004 ± 0.01	0.01 ± 0.01	*
Basophils (10^9 cells/L)	8	0.01 ± 0.02	0.08 ± 0.18	0.064
	19	0.02 ± 0.02	0.03 ± 0.01	ns
	1	0.19 ± 0.10	0.33 ± 0.27	**
Basophils (%)	8	0.14 ± 0.10	0.84 ± 1.81	0.061
	19	0.22 ± 0.12	0.21 ± 0.12	ns
	1	0.012 ± 0.008	0.006 ± 0.007	*
LUC (10^9 cells/L)	8	0.11 ± 0.11	0.11 ± 0.08	ns
	19	0.23 ± 0.15	0.18 ± 0.09	ns
	1	0.46 ± 0.30	0.26 ± 0.24	*
LUC (%)	8	1.47 ± 0.97	1.45 ± 0.90	ns
	19	2.61 ± 1.77	1.56 ± 0.79	*
CRP (ng/mL)	19	2285 ± 1503	2057 ± 1725	ns
**Lymphocyte subsets**				
	1	46.77 ± 25.8	28.68 ± 20.0	*
T-cells (% of total lymphocytes)	8	38.96 ± 27.8	39.30 ± 31.5	ns
	19	39.37 ± 33.6	26.31 ± 8.83	ns
	1	36.02 ± 9.6	82.02 ± 21.3	0.071
$CD4^+$ helper T-cells (% of total T-cells)	8	40.26 ± 16.1	165.7 ± 202	ns
	19	43.59 ± 12.3	68.42 ± 10.4	ns
	1	8.91 ± 5.1	16.20 ± 5.7	ns
$CD8^+$ cytotoxic T-cells (% of total T-cells)	8	5.47 ±2.5	15.52 ± 12.2	***
	19	5.98 ± 2.5	8.42 ± 1.7	ns
**Phagocytosis**				
	1	56.50 ± 11.02	0.07 ± 0.02	***
Neutrophil phagocytic rate (%)	8	18.32 ± 10.64	0.76 ± 0.04	***
	19	14.33 ± 11.16	0.04 ± 0.03	***
	1	23,877 ± 1216	5,647 ± 393	***
Neutrophil phagocytic capacity	8	27,792 ± 1621	5,001 ± 685	***
	19	25,368 ± 1031	4,344 ± 317	***
**Gut endpoints**				
Proximal villus height (μm)	19	470 ± 84.65	372 ± 120	**
Proximal crypt depth (μm)	19	85.68 ± 17.01	74.59 ± 14.77	*
Mid villus height (μm)	19	415 ± 88.62	412 ± 104	ns
Mid crypt depth (μm)	19	80.73 ± 9.11	72.78 ± 10.54	**
Distal villus height (μm)	19	388 ± 85.88	368 ± 71.41	ns
Distal crypt depth (μm)	19	79.77 ± 13.81	71.26 ± 8.91	*
Goblet cell density (%)	19	5.67 ± 1.68	8.29 ± 4.35	**
Lactase (U/g)	19	24.80 ± 14.60	31.71 ± 19.36	ns
Sucrase (U/g)	19	2.93 ± 1.78	5.23 ± 3.70	**
Maltase (U/g)	19	9.10 ± 4.79	13.05 ± 8.12	0.076
ApN (U/g)	19	5.07 ± 1.56	5.95 ± 2.58	ns
ApA (U/g)	19	2.76 ± 1.28	4.74 ± 2.22	***
DPP4 (U/g)	19	2.77 ± 1.05	3.74 ± 1.50	*
**Hexose absorption**				
Galactose (μmol/L)	19	265 ± 164	120 ± 128	*
**Microbial metabolites**				
Acetic acid, C2 (mmol/L)	19	39.53 ± 15.18	20.70 ± 13.51	**
Propanoic acid, C3 (mmol/L)	19	3.41 ± 2.06	2.67 ± 2.26	ns
Butanoic acid, C4 (mmol/L)	19	12.50 ± 8.41	7.93 ± 6.21	ns
Valeric acid, C5 (mmol/L)	19	0.21 ± 0.27	1.36 ± 2.88	0.073
ΣC2-5 (mmol/L)	19	53.92 ± 25.60	32.67 ± 21.42	*
3-methyl butanoic acid	19	5.51 ± 5.20	8.51 ± 10.39	ns
Propanediol (C_3OH_2), (mmol/L)	19	5.61 ± 4.85	2.79 ± 3.15	ns
Lactate (mmol/L)	19	37.23 ± 44.48	2.69 ± 5.05	*
**Acquisition of neuromuscular control**				
Eyelid opening (hours after birth)	1-3	24.98 ± 27.62	59.52 ± 65.64	**

First stand (hours after birth)	1-2	34.32 ± 27.89	43.66 ± 56.81	*
First walk (hours after birth)	1-3	37.08 ± 28.04	48.33 ± 57.17	*
**Physical activity**				
Activity (% per hour)	1-5	13.72 ± 6.03	12.39 ± 4.74	ns
Activity (% per hour)	6-8	23.88 ± 6.47	20.56 ± 6.61	0.061
**Open field**				
Distance moved (cm)	9	1810 ± 746	2041 ± 1385	ns
Velocity (cm/s)	9	10.1 ± 4.1	11.1 ± 7.5	ns
**T-maze**				
Correct choices, forward phase (%)	13-17	72.00 ± 16.75	70.34 ± 17.03	ns
Correct choices, reversal phase (%)	17-18	46.96 ± 22.25	41.43 ± 21.30	ns

[A]Measured in plasma. Relative values correspond to weight of organ relative to body weight and weight of brain region relative to total brain weight. ALAT, alanine aminotransferase; ApA, aminopeptidase A; ApN, aminopeptidase N; AST, aspartate transaminase; BASP, alkaline phosphatase; BUN, blood urea nitrogen; CRP, C-reactive protein; DPP4, dipeptidyl peptidase 4; GGT, gamma-glutamyl transferase; GLP-1, glucagon-like peptide-1; IGF-1, insulin-like growth factor-1; LUC, large unstained cells; MCHC, mean corpuscular hemoglobin concentration; MCV, mean cell volume; MFI, median fluorescent intensity; MPC, mean platelet component; MPV, mean platelet volume. p = 0.058, 0.059, 0.099, 0.051, 0.076, 0.086, 0.094, 0.097, 0.056, 0.053, 0.064, 0.061, 0.071, 0.073 indicate tendencies to a difference between groups. * $p < 0.05$; ** $p < 0.01$; ***; $p < 0.001$; ns: not significant.

Table S3

Table S3. Biochemical parameters in serum at Day 8 and 19 in preterm pigs fed bovine milk diets supplemented with WPC with high (HIGH-ALPHA) or standard α-Lac content (STANDARD-ALPHA) (mean ± SD, n = 17-18 in each group).

	**Day 8**			**Day 19**		
	**HIGH-ALPHA**	**STANDARD-ALPHA**	p	**HIGH-ALPHA**	**STANDARD-ALPHA**	p
Albumin (g/L)	12.7 ± 1.6	11.5 ± 1.7	*	20.5 ± 4.3	20.0 ± 2.5	ns
Total protein (g/L)	27.6 ± 3.0	26.4 ± 3.0	ns	34.3 ± 8.0	33.0 ± 4.0	ns
BASP (U/L)	1185 ± 472	1310 ± 418	ns	1263 ± 282	1077 ± 391	ns
ALAT (U/L)	25.0 ± 8.3	21.1 ± 5.8	ns	43.7 ± 9.3	40.1 ± 6.8	ns
Total bilirubin (µmol/L)	5.93 ± 2.6	4.79 ± 1.9	ns	1.07 ± 1.0	0.92 ± 0.9	ns
Cholesterol (mmol/L)	2.73 ± 0.6	2.74 ± 0.4	ns	2.93 ± 0.5	2.88 ± 0.7	ns
Creatinine (umol/L)	49.1 ± 8.8	46.7 ± 3.9	ns	49.9 ± 9.8	44.2 ± 7.5	*
Creatine kinase (U/L)	72.4 ± 73.5	31.0 ± 15.8	*	271 ± 239	577 ± 716	ns
Iron (µmol/L)	4.90 ± 3.4	4.91 ± 2.7	ns	6.31 ± 5.4	6.68 ± 5.9	ns
Phosphate (mmol/L)	2.06 ± 0.2	1.93 ± 0.2	0.07	2.65 ± 0.6	2.53 ± 0.3	ns
AST (U/L)	31.1 ± 15	22.6 ± 7.8	0.09	43.3 ± 16	53.9 ± 38.4	ns
BUN (mmol/L)	3.00 ± 1.8	1.33 ± 0.8	***	8.19 ± 2.6	7.00 ± 2.7	ns
GGT (U/L)	18.6 ± 8.1	19.6 ± 9.3	ns	18.6 ± 7.0	19.8 ± 6.1	ns
Calcium (mmol/L)	2.71 ± 0.1	2.74 ± 0.2	ns	3.12 ± 0.5	2.93 ± 0.3	ns
Magnesium (mmol/L)	0.84 ± 0.1	0.82 ± 0.1	ns	1.10 ± 0.3	1.06 ± 0.2	ns
Sodium (mmol/L)	148 ± 5.5	148 ± 5.6	ns	158 ± 28	145 ± 11	ns
Potassium (mmol/L)	4.81 ± 0.7	4.80 ± 0.6	ns	6.09 ± 0.8	5.48 ± 0.5	*
Lactate (mmol/L)	3.04 ± 2.5	2.22 ± 1.6	ns	9.09 ± 3.4	8.46 ± 3.1	ns
Glucose (mmol/L)	3.23 ± 0.6	3.51 ± 1.1	ns	4.95 ± 1.3	5.08 ± 1.1	ns
Insulin[A] (mU/L)	-	-	-	2.31 ± 0.02	2.98 ± 1.79	0.07
GLP-1[A] (pmol/L)	-	-	-	8.78 ± 12.02	10.76 ± 12.53	ns
Cortisol[A] (ng/ml)	-	-	-	31.76 ± 19.14	44.84 ± 78.03	ns
Serotonin[A]	-	-	-	426.86 ± 498.55	460.96 ± 486.19	ns
IGF-1[A]	-	-	-	9.86 ± 4.83	9.43 ± 4.34	ns
CRP (ng/mL)	-	-	-	2418 ± 1650	2144 ± 1367	ns

[A] Measured in plasma. ALAT, alanine aminotransferase; AST, aspartate transaminase; BASP, alkaline phosphatase; BUN, blood urea nitrogen; CRP, C-reactive protein; GGT, gamma-glutamyl transferase; GLP-1, glucagon-like peptide-1; IGF-1, insulin-like growth factor-1. p = 0.07 and 0.09 indicate tendencies to a difference between groups. * $p < 0.05$; ***; $p < 0.001$; ns: not significant.

Table S4

Table S4. Hematology, lymphocyte subsets and phagocytosis analyzed in blood collected at birth, at Day 8 and 19 in preterm pigs fed a bovine milk diet supplemented with WPC with high (HIGH-ALPHA) or standard α-Lac content (STANDARD-ALPHA) (mean ± SD, n = 19 in each group).

	Day	HIGH-ALPHA	STANDARD-ALPHA	*p*
**Hematology**				
	1	3.87 ± 0.3	4.06 ± 0.4	ns
Erythrocytes (10^{12} cells/L)	8	3.69 ± 0.6	3.74 ± 0.4	ns
	19	4.13 ± 0.4	4.25 ± 0.6	ns
	1	5.15 ± 0.4	5.42 ± 0.4	ns
Hemoglobin (mmol/L)	8	4.45 ± 0.6	4.51 ± 0.5	ns
	19	4.01 ± 1.0	4.51 ± 0.6	ns
	1	0.29 ± 0.03	0.44 ± 0.6	ns
Hematocrit (L/L)	8	0.25 ± 0.03	0.41 ± 0.6	ns
	19	0.39 ± 0.6	0.27 ± 0.04	ns
	1	76.4 ± 4.9	76.5 ± 4.4	ns
MCV (fL)	8	67.0 ± 2.0	68.1 ± 3.2	ns
	19	61.6 ± 3.3	63.0 ± 4.4	ns
	1	17.5 ± 0.8	17.52 ± 0.6	ns
MCHC (mmol/L)	8	17.6 ± 0.3	17.8 ± 0.6	ns
	19	16.1 ± 3.9	17.0 ± 0.5	ns
	1	295 ± 34	324 ± 43	ns
Platelets (10^9 cells/L)	8	436 ± 182	461 ± 114	ns
	19	656 ± 127	753 ± 283	ns
	1	11.2 ± 1.2	10.4 ± 1.1	*
MPV (fL)	8	14.0 ± 4.5	11.4 ± 2.7	*
	19	13.4 ± 4.4	11.0 ± 2.7	*
	1	230 ± 5.7	230 ± 6.6	ns
MPC (g/L)	8	246 ± 7.02	242 ± 7.8	ns
	19	244 ± 7.1	239 ± 11	ns
**Immune cell counts**				
	1	2.55 ± 0.4	2.65 ± 0.4	ns
Leukocytes (10^9 cells/L)	8	6.90 ± 2.6	5.81 ± 2.1	ns
	19	9.21 ± 3.5	9.44 ± 3.4	ns
	1	0.57 ± 0.2	0.55 ± 0.2	ns
Neutrophils (10^9 cells/L)	8	5.64 ± 4.04	4.00 ± 2.0	ns
	19	5.05 ± 2.9	5.34 ± 3.3	ns
	1	22.2 ± 5.06	21.1 ± 5.7	ns

Neutrophils (%)	8	72.6 ± 8.82	66.5 ± 14.0	ns
	19	51.0 ± 15.5	52.3 ± 14	ns
	1	1.91 ± 0.03	2.01 ± 0.4	ns
Lymphocytes (10^9 cells/L)	8	1.57 ± 0.7	1.53 ± 0.7	ns
	19	3.14 ± 1.2	3.13 ± 0.8	ns
	1	74.9 ± 5.3	75.9 ± 6.5	ns
Lymphocytes (%)	8	23.7 ± 7.85	29.0 ± 13.7	ns
	19	37.5 ± 15.02	37.2 ± 12	ns
	1	0.04 ± 0.02	0.04 ± 0.02	ns
Monocytes (10^9 cells/L)	8	0.12 ± 0.09	0.16 ± 0.2	ns
	19	0.66 ± 0.4	0.62 ± 0.4	ns
	1	1.75 ± 0.7	1.37 ± 0.6	0.08
Monocytes (%)	8	1.74 ± 1.02	2.55 ± 1.9	ns
	19	7.24 ± 3.9	6.73 ± 3.4	ns
	1	0.01 ± 0.01	0.03 ± 0.03	ns
Eosinophils (10^9 cells/L)	8	0.02 ± 0.02	0.02 ± 0.02	ns
	19	0.07 ± 0.1	0.07 ± 0.1	ns
	1	0.50 ± 0.3	0.98 ± 1.2	ns
Eosinophils (%)	8	0.36 ± 0.2	0.34 ± 0.2	ns
	19	0.73 ± 0.9	0.63 ± 0.9	ns
	1	0.00 ± 0.0	0.0 ± 0.01	ns
Basophils (10^9 cells/L)	8	0.01 ± 0.01	0.01 ± 0.03	ns
	19	0.02 ± 0.01	0.02 ± 0.02	ns
	1	0.20 ± 0.1	0.18 ± 0.1	ns
Basophils (%)	8	0.14 ± 0.09	0.14 ± 0.1	ns
	19	0.21 ± 0.2	0.24 ± 0.1	ns
	1	0.01 ± 0.01	0.01 ± 0.01	ns
LUC (10^9 cells/L)	8	0.10 ± 0.08	0.12 ± 0.1	ns
	19	0.28 ± 0.1	0.27 ± 0.2	ns
	1	0.46 ± 0.3	0.50 ± 0.3	ns
LUC (%)	8	1.46 ± 1.0	1.48 ± 1.0	ns
	19	3.35 ± 2.0	2.89 ± 1.6	ns
**Lymphocyte subsets**				
	1	44.5 ± 27.2	47.7 ± 25.5	ns
T-cells (% of total lymphocytes)	8	39.9 ± 29.9	38.1 ± 27.7	ns
	19	38.5 ± 37.0	46.3 ± 36.1	ns

	1	36.1 ± 9.1	37.3 ± 10.7	ns
CD4+ helper T-cells (% of total T-cells)	8	40.4 ± 16.2	40.1 ± 17.7	ns
	19	40.6 ± 9.5	36.6 ± 6.2	ns
	1	9.36 ± 4.6	8.71 ± 5.4	ns
CD8+ cytotoxic T-cells (% of total T-cells)	8	5.02 ± 2.5	5.92 ± 2.75	ns
	19	4.77 ± 1.3	5.35 ± 1.2	ns

LUC, large unstained cells; MCHC, mean corpuscular hemoglobin concentration; MCV, mean cell volume; MFI, median fluorescent intensity; MPC, mean platelet component; MPV, mean platelet volume; NK cells, natural killer cells. $p = 0.08$ indicates a tendency to a difference between groups. * $p < 0.05$; ns: not significant.
